# Revealing structural peculiarities of homopurine GA repetition stuck by i-motif clip

**DOI:** 10.1093/nar/gkab915

**Published:** 2021-10-28

**Authors:** Aleš Novotný, Jan Novotný, Iva Kejnovská, Michaela Vorlíčková, Radovan Fiala, Radek Marek

**Affiliations:** CEITEC – Central European Institute of Technology, Masaryk University, Kamenice 5, CZ-62500 Brno, Czechia; National Centre for Biomolecular Research, Faculty of Science, Masaryk University, Kamenice 5, CZ-625 00 Brno, Czechia; CEITEC – Central European Institute of Technology, Masaryk University, Kamenice 5, CZ-62500 Brno, Czechia; National Centre for Biomolecular Research, Faculty of Science, Masaryk University, Kamenice 5, CZ-625 00 Brno, Czechia; Institute of Biophysics of the Czech Academy of Sciences, Královopolská 135, CZ-612 65 Brno, Czechia; Institute of Biophysics of the Czech Academy of Sciences, Královopolská 135, CZ-612 65 Brno, Czechia; CEITEC – Central European Institute of Technology, Masaryk University, Kamenice 5, CZ-62500 Brno, Czechia; National Centre for Biomolecular Research, Faculty of Science, Masaryk University, Kamenice 5, CZ-625 00 Brno, Czechia; CEITEC – Central European Institute of Technology, Masaryk University, Kamenice 5, CZ-62500 Brno, Czechia; National Centre for Biomolecular Research, Faculty of Science, Masaryk University, Kamenice 5, CZ-625 00 Brno, Czechia

## Abstract

Non-canonical forms of nucleic acids represent challenging objects for both structure-determination and investigation of their potential role in living systems. In this work, we uncover a structure adopted by GA repetition locked in a parallel homoduplex by an i-motif. A series of DNA oligonucleotides comprising GAGA segment and C_3_ clip is analyzed by NMR and CD spectroscopies to understand the sequence–structure–stability relationships. We demonstrate how the relative position of the homopurine GAGA segment and the C_3_ clip as well as single-base mutations (guanine deamination and cytosine methylation) affect base pairing arrangement of purines, i-motif topology and overall stability. We focus on oligonucleotides C_3_GAGA and methylated GAGAC_3_ exhibiting the highest stability and structural uniformity which allowed determination of high-resolution structures further analyzed by unbiased molecular dynamics simulation. We describe sequence-specific supramolecular interactions on the junction between homoduplex and i-motif blocks that contribute to the overall stability of the structures. The results show that the distinct structural motifs can not only coexist in the tight neighborhood within the same molecule but even mutually support their formation. Our findings are expected to have general validity and could serve as guides in future structure and stability investigations of nucleic acids.

## INTRODUCTION

DNA can, depending on the primary sequence, adopt various secondary structures distinctly different from the classical WC double helix. These non-canonical structures frequently appear in important regions of the genome and have specific functions in regulating biological processes (1). Actually, searching for new sequence-dependent conformational arrangements, understanding principles of their formation and functional consequences are prerequisite for the possibility of controlling gene expression and for the rational use of scientific findings in medical applications (2).

Alternating d(GA)_n_·d(TC)_n_ sequences are abundant in genomes (3–6). They are especially frequent in genomes of rodents and primates, where a significant fraction of them is found in long (*n* ≥ 30 base-pairs) blocks (3). This microsatellite sequence is extremely polymorphic (7,8). Except for classical duplex and triplexes, the particular single strands can adopt various non-canonical arrangements: the d(CT) strand can form an intercalated iM with thymine bulges in slightly acidic conditions, and numerous models were proposed for the arrangements of the d(GA) sequence strand: alpha helix-like ordered single strands, parallel and also antiparallel duplexes, and tetraplexes (8–11). The polymorphism of the sequence may play a role in a plenty of their different biological functions reported: Microsatellite d(GA)·d(TC) is a recombination hot spot. A significant enhancement of homologous DNA recombination in mini-chromosomes of SV40 polyomavirus was found in humans and monkeys (12). It has been shown that the PGB protein found in human fibroblast cells selectively binds single stranded d(GA) repeats, induces strand separation of the WC paired heteroduplex and stabilizes formation of triple helices or other unusual DNA structures (13). The sequence d(AG)_4_ was found to have a higher activity as a primer for DNA polymerase I in *Escherichia coli* than any other dinucleotide repeat (14). Other experimental studies have shown that d(GA)_*n*_ repetitions play various roles in different genes, e.g. at hsp26 promoter of *Drosophila melanogaster* the d(AG) sequences surround the region responsible for the nucleosome binding (15) and have a critical influence on the formation of DNase I hypersensitive sites (16).

Self-association of d(GA)_*n*_ has been studied since 1980s when its tetrahelical arrangement was proposed at neutral pH (17,18). Another work employing pH titrations that induced disproportion of poly[d(AG)·d(CT)] sequence monitored by CD distinguished six rearrangements (duplex → triplex + free d(AG) → ‘acid-induced self-complex’ of purine strand even in the presence of a complementary pyrimidine strand) (19). Similarly, the secondary structure of d(GA)_10_ at a lower salt (<10 mM Na^+^) concentration and acidic pH was described as a single-stranded left-handed helix built form unstacked nucleobases with sequential intramolecular ionic bonds between protonated A and the phosphate group of G (8,20). It was proved by CD experiments that such ordered d(GA)_*n*_ single strands tend to dimerize into homoduplex at higher salt concentrations without substantial conformational rearrangement (21). Several spectroscopic and chemical-probing experiments provided data analogous to those of parallel G-quadruplexes suggesting that the d(GA)_10_ oligonucleotide at high salt concentrations (≥100 mM Na^+^) of uni-univalent salts (or more potent MgCl_2_) adopts a parallel duplex with direct stacking of G·G base-pairs (bps) and the intervening A residues pushed outside the duplex (22). Rippe *et al.* (9) proposed a parallel double helix structure of the d(GA)_n_ formed by symmetric *syn* G·G bps in N1H-O6 geometry (associated via Watson–Crick edge, WCE) and *anti* A·A bps in N6H-N7 geometry (Hoogsteen edge, HE), *vide infra*.

Interestingly, the single 5′-GA-3′ segment in parallel duplexes adopts base pairing geometry different to the ones proposed above. In the work by Robinson *et al.* (23), it was suggested that at acidic pH, CGATCG adopts a parallel duplex structure with the G^2^·G^2^ bp in N2H-N3 geometry (sugar edge, SE) and A^3^·A^3^ bp in HE geometry. A similar pairing pattern was found in sequences (CGA)_2_C, C(GA)_3_, C(GA)_3_C where GA segment is attached to CH^+^·C pair at its 5′-end (24,25). The unusual compact pairing of guanine was recognized, among others, based on substantial shielding of N1H imino proton and inter-strand NOE connectivities observed in ^1^H NMR spectra. This specific arrangement (termed as GA step in this paper) was found to be a powerful motif in promoting parallel duplex formation. An extraordinary stability of this structure was ascribed to the effective inter-strand stacking overlap as shown in detail in the structure of d(TCGA) duplex determined using NMR spectroscopy (see Supplementary Figure S1) (26).

To investigate the structure of d(GA)_n_ dinucleotide repeats arranged in parallel duplex (27) and consequently clarify, in some cases, contradicting claims about stoichiometry, protonation state of adenine and type of base pairing found in the literature (18–21), a robust and well-defined model sequence is essential. We started our investigation with d(GA)_*n*_ (*n* = 5, 10) sequences. However, the broad signals in their NMR spectra indicated dynamic ensemble of structures preventing the precise structure determination (see Supplementary Figure S2). Therefore, the flexibility of system was decreased by shortening the d(GA)_*n*_ block to two repeats (*n* = 2). To impose parallel orientation of the strands in our models, we introduced a C_3_ segment forming an i-motif (iM). A similar strategy was employed to stabilize a parallel duplex composed of A-T bps in reverse WC geometry (28). The resulting tremendous improvement in spectral properties encouraged us to design a series of constructs with different relative positions of the homopurine part and the iM clip, which allowed the high-resolution structures to be determined, *videinfra*.

The iM is constituted from hemi-protonated cytosine bp (CH^+^·C) formed under acidic conditions (29,30). Recent papers, however, show that, with increasing length of C-rich sequence (31,32), formation of the iM can shift to the neutral pH range. The interest of scientific community in iMs has been spurred by recent demonstrations of their existence *in vivo*. Specifically, the iM structures were detected in regulatory regions of the human genome, namely in promoters and telomeric regions by iM-specific fluorescently marked antibody (33). In the same time, the existence of an iM formed by selected promoter sequences was demonstrated by in-cell NMR experiments (34). Recently, the compatibility of iM with B-DNA at neutral pH has been demonstrated (35). In that work, the unimolecular iM is stabilized by a hairpin at one side and a minor groove tetrad at the other side. In general, the iM structure can adopt two topologies differing in the intercalation pattern: in the 3′*E* arrangement, the outermost CH^+^·C bp is located at 3′-end of the sequence, whereas 5′*E* arrangement is characterized by a 5′-external CH^+^·C bp (36). Previously, the overall higher stability of extended 3′*E* topology was attributed to a larger number of favourable weak CH···O4′ hydrogen bonds between sugar moieties as indicated by a comparative MD simulation (37). This preference can be inverted if the resulting structure is a kinetic product of the folding process (38). Incorporation of 5-methylcytosine (mC) was reported to induce thermal stabilization of iMs and to modulate the preferred topology (39,40). Here, we used mC-for-C substitution as a label facilitating the assignment of NMR signals of the iM.

In this work, we performed a systematic CD and NMR investigation of the stability and structure of various d(C_3_R_4_) and d(R_4_C_3_) oligonucleotides where R_4_ stands for GA or AG repeat. Their global fold was examined using CD, absorption and 1D ^1^H NMR spectra. For sequences adopting well-defined structures, NMR data were used to build high-resolution models that were further refined and analysed based on MD simulations in the explicit solvent. The goals of this study are addressed in the following sequence:

- To screen a global structure and stability of designed sequences by using CD and absorption spectroscopy
- To determine iM topology and base pairing of the purine block in various sequential contexts based on ^1^H NMR experiments
- To characterize the modulation of iM topology by 5-methylation of cytosine
- To describe the structural differences in R-C and C-R steps using high-resolution models and to correlate these changes with global stability

## MATERIALS AND METHODS

### Preparation of oligonucleotide samples

All oligonucleotides (see Table 1) were synthetized and HPLC-purified by supplying company Merck. Dried pellets were dissolved in 500 μl of solution containing 10 mM potassium phosphate and 50 mM KCl (total of 65 mM K^+^) at pH 5. All measurements were performed in this solution, unless stated otherwise. For NMR measurements, the oligonucleotide samples were subjected to 4 cycles of centrifugal filtration using 3 kDa milipore filters (AMICON) and concentrated to 50 μl. Afterwards, 50 μl of D_2_O and 400 μl of above-described solution were added to yield 500 μl of final solution which was annealed overnight followed by a check of pH. Selected samples were lyophilized and transferred to 99.95% D_2_O.

**Table 1.** The oligonucleotide sequences analysed in this work. Melting temperatures (*T*_m_) were determined by monitoring changes of Δϵ at wavelengths of dominating CD bands. The presence of hysteresis (*H*) was assessed by UV absorption melting and renaturation experiments (see Supplementary Figure S3). The measurements were performed in 10 mM potassium phosphate and 50 mM KCl (65 mM K^+^), pH 5, at 0.15 mM strand concentration.

Sequence (5′→ 3′)^a^										*T*_m_ [°C]^b^	λ [nm]	*H* [°C]^c^	λ [nm]
_1_	_2_	_3_	_4_	_5_	_6_	_7_	_8_	_9_	_10_	CD detection		UV abs. detection	
C	C	C	G	A	G	A				37	263	N	295
_m_C	C	C	G	A	G	A				44	262	N	306
C	C	_m_C	G	A	G	A				39	263	S	305
C	C	C	I	A	G	A				21^d^	261	**M**	297
C	C	C	G	A	I	A				33^d^	264	N	297
C	C	C	G	A	G	A	G			37	263	N	297
C	C	C	G	A	G	A	G	A	G	39	264	N	297
C	C	C	A	G	A	G				18	261	S	295
G	A	G	A	C	C	C				39	285	**L**	295
G	A	G	A	_m_C	C	C				50	285	S	305
G	A	G	A	C	C	_m_C				48	285	**L**	303
A	G	A	G	C	C	C				27	285	S	297
A	G	A	G	_m_C	C	C				32	292	S	304
A	G	A	G	C	C	_m_C				34	286	S	305
C	C	C								n.d.^e^	289		
C	C	C	T	T	T	T				14	283		

^a^5-methylcytidine and inosine are abbreviated _m_C and I, respectively, in our base-modified nucleotides.

^b^The estimated error in *T*_m_ is ± 1°C.

^c^Hysteresis (*H*): (N, none) *H* < 1°C, (S, small) *H* < 5°C, (**M**, medium) *H* < 10°C, (**L**, large) *H* > 10°C. More details and commentary on hysteresis are given in Supplementary Figure S3.

^d^The dramatic effect of deamination (G→I, particularly for C_3_IAGA) is discussed in relation to the base pairing in the purine stretch in section Structure of purine homoduplex.

^e^n.d., not determined (*T*_m_ < 10°C).

### UV absorption and CD spectroscopy

DNA strand concentrations were determined based on UV absorption measured at 260 nm on a UNICAM 5625UV/Vis spectrophotometer (Cambridge, U.K.) using molar absorption coefficients calculated by the nearest neighbour method (41). Sample concentration for CD experiments was (unless stated otherwise) 0.15 mM per strand.

CD measurements were carried out using a Jasco 815 (Tokyo, Japan) dichrograph in 0.05 cm (singularly in 0.02 cm) path-length quartz Hellma cells placed in a Peltier cell holder. Spectra were measured in the range of 220–330 nm with scan speed 100 nm/min and a set of four scans was averaged for each spectrum. CD signal was expressed as a difference in the molar absorption Δϵ of the left- and right-handed circularly polarized light, according to the formula Δϵ [M^–1^cm^–1^] = *θ* / (32.98 *c l*), where *θ* is the measured ellipticity value [mdeg], *c* is the molar strand concentration [M], and *l* is the optical path of a used cell [cm] (41).

CD melting experiments were monitored by changes in Δϵ at wavelengths corresponding to dominating CD band of particular sequences (specified in Table 1 and figures). The temperature was increased in 2°C steps and the samples were equilibrated for 2 min at each temperature before collecting the spectrum (the total time of keeping at each temperature point was ∼6 min resulting in the rate of temperature changes ∼0.33°C/min). The *T*_m_ values were determined from dual baseline-corrected 1 - 0 normalized curves (1- native and 0- denatured forms) as temperatures, at which half of the molecules were folded (42). The error associated with determination of melting temperature was estimated to be ± 1°C based on repeated measurements.

Hysteresis between melting and renaturation processes were checked using UV absorption measured on a Varian Cary 4000 (Mulgrave, Australia) spectrometer and determined as a difference between melting (*T*_m_) and renaturation (*T*_ren_) temperature. The absorption melting and denaturation experiments were carried out at 0.15 mM DNA concentration using 0.05 cm cells at three rates of temperature changes: in 1°C increments with 3 and 6 min waiting prior to taking each spectrum (total time 4.5 and 7.5 min per point), resulting in ∼0.22 and 0.13°C/min, respectively and also in 2°C increments and 4.5 min waiting at each temperature (total time 6 min per point) resulting in 0.33°C/min as in the CD measurement. The *T*_m_ and *T*_ren_ values were determined as stated above for CD melting experiments. The course of melting and renaturation experiments taken in 10 mM potassium phosphate and 50 mM KCl, pH 5 (adjusted by 0.1 M HCl) was compared for selected sequences with the melting experiments carried out in solution of potassium Robinson-Britton buffer (K-RB), pH 5 with added KOH up to the final concentration 65 mM K^+^). K-RB buffer was prepared from a mixture of 0.04 M acids (boric, phosphoric and acetic) and 0.2 M KOH.

### NMR spectroscopy

The ^1^H 1D and 2D NMR spectra of studied sequences were measured on Bruker Avance III HD 600 and 700 MHz spectrometers equipped with quadruple-resonance cryoprobe and triple-resonance room temperature probes, respectively. The NMR samples were prepared at 0.2–1.5 mM strand concentration in 90%/10% H_2_O/D_2_O. The strand concentrations of all NMR samples are specified in captions of figures of corresponding NMR spectra. The purity and uniformity of samples was evaluated by using 1D ^1^H NMR spectra at 2–5°C and 25°C. For well-folded samples, 2D ^1^H-^1^H NOESY spectra were recorded with 200 ms mixing time. WATERGATE (43) pulse sequence was used for the solvent signal suppression. Sets of 2D ^1^H-^1^H NOESY (44,45) experiments were recorded on selected samples in 99.95% D_2_O at 70, 100 and 150 ms mixing time to determine interproton distances. Resolved ^31^P shifts were assigned based on ^1^H-^31^P COSY experiment (46). Acquired data were processed using TopSpin3.2 software. Sparky 3.114 (47) was used for the resonance assignment and integration of NOE cross-peaks.

### Molecular modelling

All molecular dynamics simulations were performed in Amber 16 (48) with parmbsc1 (49) force field. Simulated annealing protocol was adopted from Amber 16 (48,50,51) reference manual. Energy minimization was performed by 1000 cycles of steepest descend and 1000 cycles of conjugate gradient algorithms. During simulated annealing we used NOE-derived distance restraints, inter-base H-bond distance restraints, χ-angle restraints, and base-pair planarity restraints. Distance restraints were obtained by extrapolation of NOE cross-peak volumes to zero mixing time and recalculated to distance using cytosine H5-H6 distance (2.48 Å) as a reference. Resulting distances were divided into three categories: strong (1.8–3.6 Å), medium (2.6–5.0 Å), and weak (3.5–6.0 Å) (similar to setup employed in reference (52)). Overlapped NOE cross-peaks, cross-peaks including exchangeable protons, and cross-peaks present only at longer mixing times were included as distance restraints with an increased range (2.5–6.5 Å). Inter-base H-bond distance restraints were obtained from DFT-optimized base-pair geometries (DFT with B3LYP functional (53,54), D3-BJ dispersion correction (55) and def2-TZVPP (56) basis set performed in the Turbomole program (57), www.turbomole.org). Additionally, we used χ-angle restraints (*syn*: 25 – 95°, *anti*(pyrimidines): 170 – 310°, *anti*(purines): 200 – 280°) and pseudo-torsion restraints to maintain base-pair planarity. Force constants of 20 kcal·mol^–1^·Å^–2^ were used for NOE-derived and H-bond restraints, 200 kcal·mol^–1^·Å^–2^ for χ-angle restraints and 100 kcal·mol^–1^·Å^–2^ for base-pair planarity restraints.

A starting structure of the homoduplex segment was built manually from nucleotides which were arranged to qualitatively respect right-handed helicity and experimental data regarding χ-torsion angle. Starting structure of iM clip was acquired from structure of C_4_A_2_ (PDB: 1YBN) (36). Isolated structures of homoduplex segment and iM clip were subjected to simulated annealing with above-described restraints. Afterwards, the homoduplex segment was manually attached on both ends of the iM clip. The energy of the resulting structure was minimized, and the minimized structure was subjected to simulated annealing with complete set of restraints. As the restrain violations between different simulated annealing runs were comparable, the most symmetrical structure was selected for further simulations. The selected structure was solvated by 12 Å layer of water with Na^+^ and Cl^–^ ions to yield isotonic concentration using *Solvate* software (www.mpibpc.mpg.de/grubmueller/solvate). Truncated octahedron was created afterward by *tleap* (48). The solvated molecule was subjected to a 10-stage temperature equilibration (58) in order to relax both explicit solvent and solute. Restraints were included in the last two stages of equilibration.

### Unbiased molecular dynamics

Constant pressure unbiased molecular dynamics simulation (MD) was performed on previously equilibrated structures at 300 K with SHAKE algorithm (59) constraining length of bonds with hydrogen. The length of integration step was set to 2 fs. Target pressure was set to 1 bar and pressure relaxation time to 6 ps. Temperature was regulated by Berendsen thermostat (60) with heat bath coupling constant of 5 ps. Non-bonded cutoff was set to 8 Å. No restraints were applied during MD. A snapshot was selected every 100 ps of MD yielding 10 000 snapshots from 1 μs of simulation. Selected structural parameters were extracted from the trajectory using *cpptraj* tool (61). Software 3DNA (62) was used to evaluate overlaps between stacked base-pairs.

## RESULTS AND DISCUSSION

**Figure 1.**
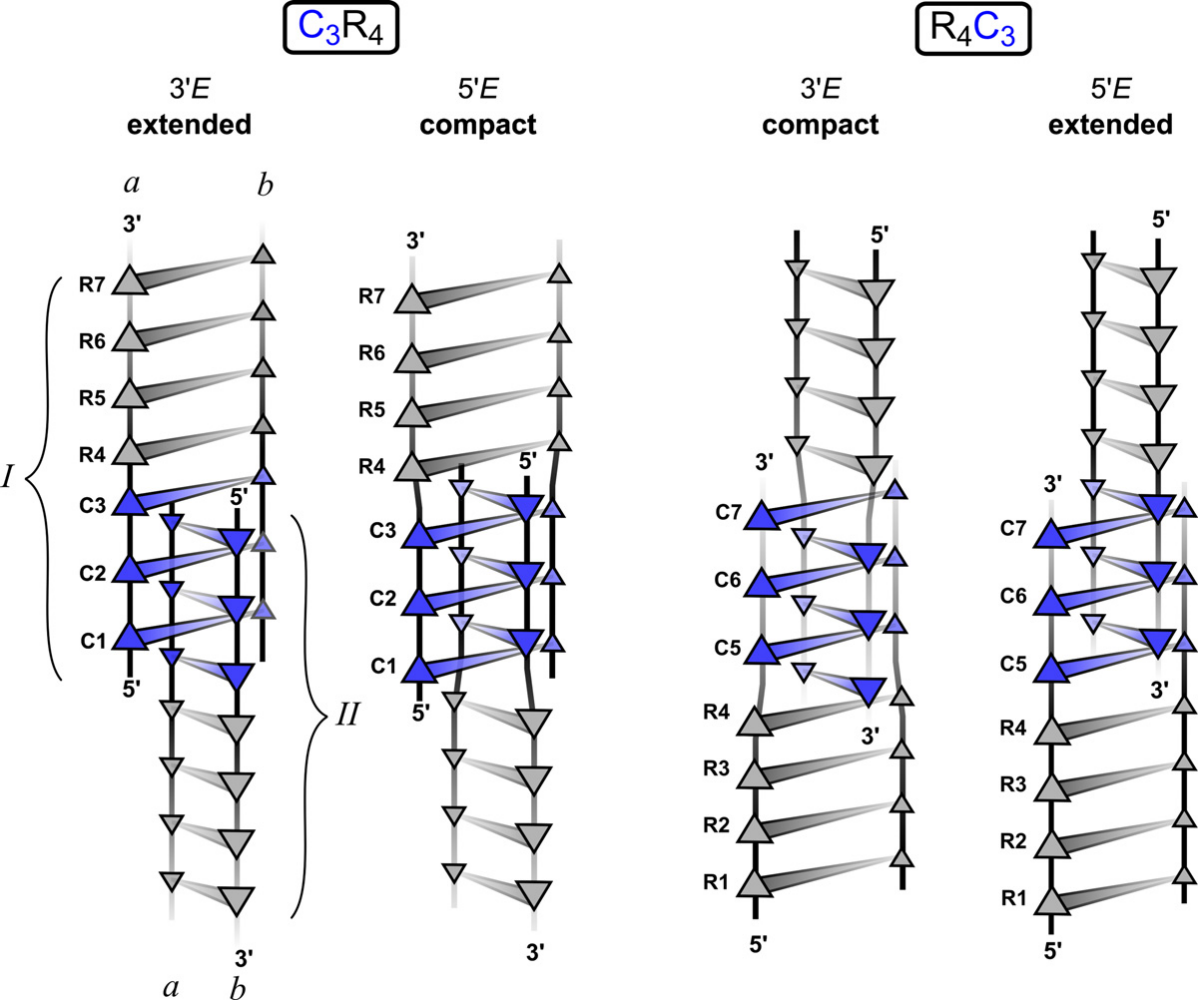
Scheme of possible C_3_R_4_ and R_4_C_3_ arrangements: Both classes of sequences (C_3_R_4_ and R_4_C_3_) studied in this work can adopt two possible iM topologies: 3′*E* arrangement with the outermost CH^+^·C bp at 3′-end, 5′*E* arrangement with the outermost CH^+^·C bp at 5′-end. The 5′→3′ polarity of individual strands is depicted by the orientation of triangles. The cytosines are denoted by blue and purines by grey triangles. The alternative descriptors reflecting the compactness of the iM clip are indicated in the header. Note the different stacking modes at C-R and R-C steps in extended and compact topology: In the extended topology the R·R bp is directly stacked on to CH^+^·C bp of the same duplex whereas in the compact topology the direct stacking is interrupted by intercalated CH^+^·C bp. The requirement to accommodate CH^+^·C bp in compact iM topologies may affect the geometry of R·R bp, *vide infra*.

We have studied purine segments GAGA and AGAG attached to the iM clip either at 5′- or 3′-end (Figure 1). In the text below we use the following nomenclature to specify residues and interatomic contacts. Because i-motif (iM) can be viewed as a pair of intercalated parallel duplexes, we use subscripts *I* and *II* to distinguish nucleotides of the two duplexes and subscripts *a* and *b* to differentiate nucleotides within a duplex. All base-pairs are formed between symmetrically equivalent residues, e.g. ${\rm{G}}_{Ia}^4$ base pairs with ${\rm{G}}_{Ib}^4$ in duplex *I*. A list of oligonucleotides studied in this work is given in Table 1.

### Global fold and stability

**Figure 2.**
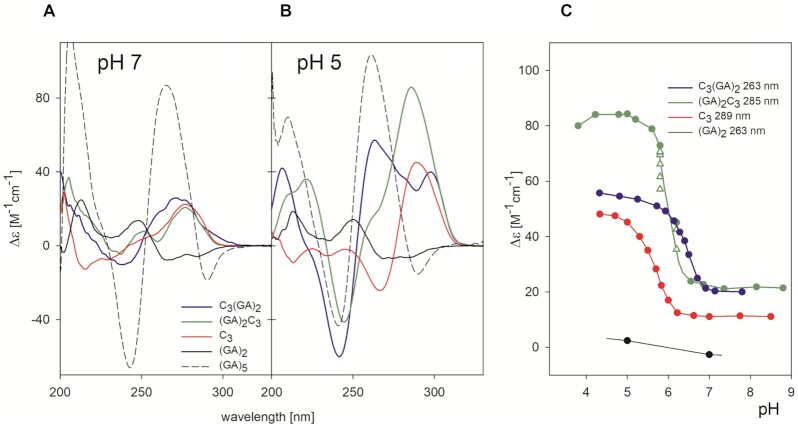
CD spectra comparing both isolated structural components (C_3_ and GA repetition) with two of the main constructs investigated in this work, C_3_GAGA and GAGAC_3_ (**A**) at pH 7 and (**B**) at pH 5. All measurements were done in 10 mM potassium phosphate and 50 mM KCl (total of 65 mM K^+^) apart from (GA)_5_ for which the 100 mM K^+^ was added (total of 115 mM K^+^) to stabilize its neutral parallel duplex. Due to the inability of (GA)_2_ to adopt any ordered secondary structure, we show the fingerprint of CD spectra of parallel duplex on the longer (GA)_5_ sequence. (**C**) pH-induced changes in CD spectra of the studied sequences monitored by Δϵ at wavelengths indicated. Open triangles correspond to non-equilibrium states. CD spectra were measured at 1°C, in 0.1 cm cells at 0.1 mM DNA strand concentration. The dependences started at alkaline pH and proceeded toward acidic pH.

The first estimation of the degree of folding for all the oligonucleotides was based on CD spectra. (GA)_*n*_ repeats with *n* ≥ 5 at neutral pH provide CD spectra characteristic for a parallel duplex (see (GA)_5_ in Figure 2A) with positive band at 260 nm and a negative band at 240 nm regardless of salt type. The same type of spectrum but containing a shallower negative band is displayed by an ordered single strand of the GA repeat formed at acidic pH (Figure 2B) or under dehydrating conditions (21). The short GAGA sequence studied in this work remains unstructured under both mentioned conditions. In contrast, the C_3_ segment associates into the tetramolecular iM at acidic pH close to the cytosine p*K*_a_ value (Figure 2B). Its CD spectrum is characteristic by a dominating positive band at 285 nm and a negative band at 265 nm. Interestingly, connecting the two short sequences, C_3_ and GAGA, gives rise to ordered structures at acidic pH with distinct positive CD amplitudes in the positions of the characteristic CD bands of the two structural components (Figure 2). The band at 260 nm dominates in the CD spectrum of C_3_GAGA, and the one at the long wavelength side is lower. In contrast, GAGAC_3_ provides a very high CD band at 285 nm and only a shoulder around 260 nm. Thus, in both cases, the shape of the CD spectrum is strongly influenced by the structural component positioned at the 3′-end of the molecule. Both, C_3_ as well as C_3_GAGA and GAGAC_3_ provide conservative, plain spectra at pH 7 indicating that no ordered structure is formed. It is the pH-driven formation of iM that enables constitution of the new structures consisting of a tetramolecular iM clip and presumed GAGA homoduplex on both of its sides (see Figure 1). Therefore, we compare CD spectra and pursue all further experiments at pH ∼ 5. However, it is to be noted that while C_3_ adopts stable structures only at pH 5 and (GA)_2_ remains unfolded, both C_3_GAGA and GAGAC_3_ transform toward their stable structures around pH 6 already (Figure 2C). This is very exceptional observation of a synergy between two structural blocks previously reported only for i-motif and G-quadruplex in a single 38 nucleotide molecule (63).

**Figure 3.**
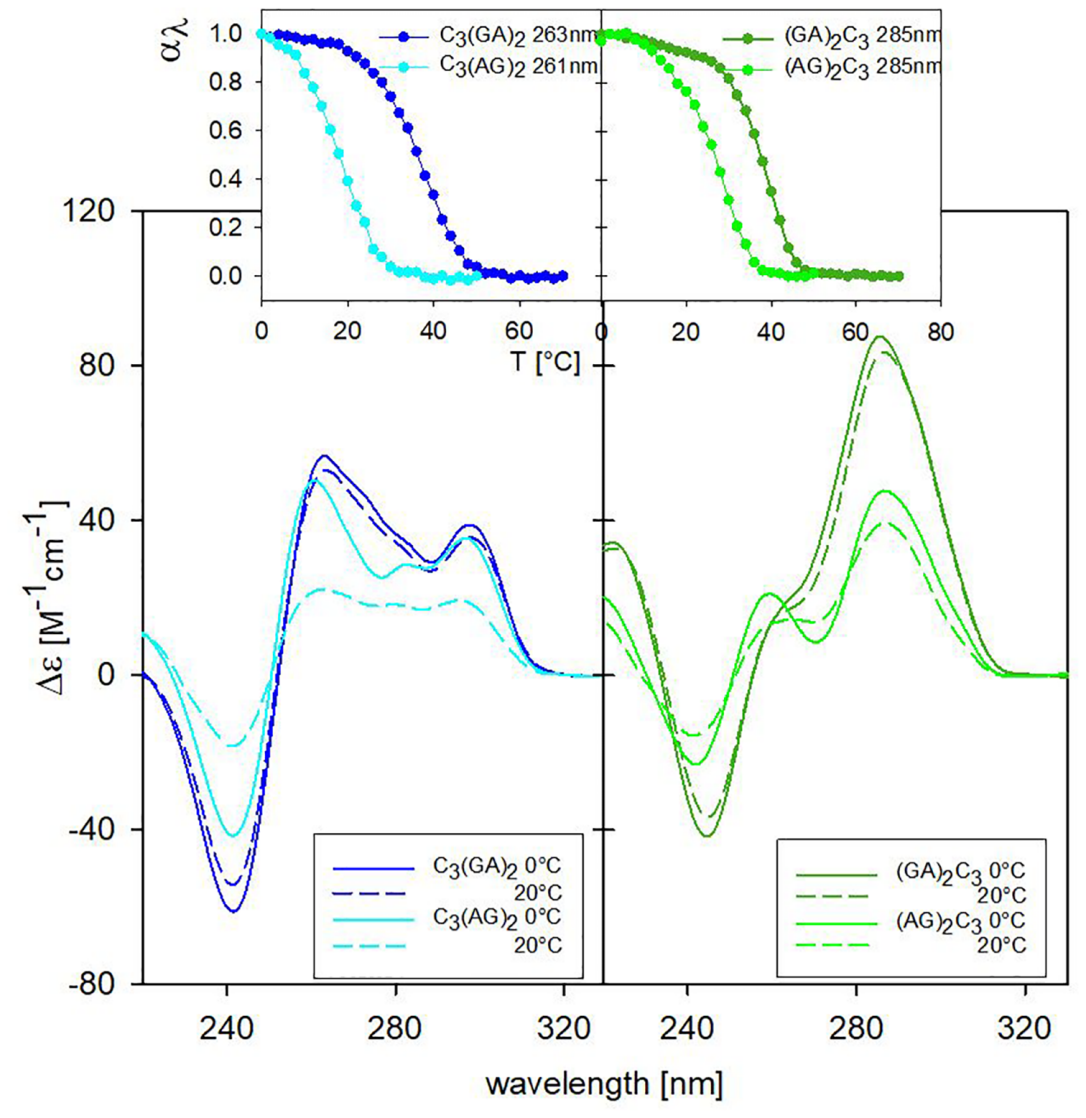
CD spectra and corresponding normalized melting curves of parental sequences C_3_GAGA/C_3_AGAG (left) and GAGAC_3_/AGAGC_3_ (right) measured at 0.15 mM concentration in 65 mM K^+^, pH 5. Both C_3_GAGA and GAGAC_3_ show similar thermostability; the analogues containing the AGAG segment instead of GAGA are less stable.

In the following experiment we compare the effect of connecting C_3_ block with GAGA and AGAG (Figure 3). Both sequences C_3_AGAG and AGAGC_3_ form ordered and cooperatively melting structures. CD spectra indicate that the structure of C_3_AGAG is similar to that of C_3_GAGA, but it is markedly less thermostable (Table 1). In contrast, the CD spectrum of AGAGC_3_ differs from that of GAGAC_3_. Its positive long-wavelength band is distinctly reduced, and a positive band at 260 nm may indicate a unique structural feature in AGAGC_3_. The structures with AGAG repetition are much less thermostable than those containing GAGA repeat.

Melting of all four sequences is fully reversible. However, whereas the courses of melting and refolding of C_3_GAGA follow the same curve, and only slightly differ in the case of C_3_AGAG, the sequence AGAGC_3_ and, especially, GAGAC_3_ display a hysteresis between the two processes (Supplementary Figure S3). The hysteresis diminished only slightly upon increasing the time for temperature equilibration. The presence of hysteresis in the case of sequences with purine block at the 5′-end indicates a slow and distinct kinetics of melting and renaturation of their structures. As the CD experiments were performed at much lower DNA concentration compared to the NMR measurements, we checked the CD spectra of the four parental sequences at distinctly increased DNA concentration approaching those used in NMR. The CD spectra of the sequences at 0.7 mM DNA concentration (Supplementary Figure S4) are principally the same as those at the concentration used for CD measurements with only slightly higher amplitudes. As expected for intermolecular assembly, the melting temperatures increase at higher oligonucleotide concentration (Supplementary Figure S4) (by 6°C on average, data not shown).

Based on the analysis of the melting curves derived from CD measurements, the stability trends of the sequences summarized in Table 1 can be verbally expressed as follows:

- Sequences containing 5′-GA-3′ repetition (i.e. C_3_GAGA and GAGAC_3_) show a similar stability regardless of their position relative to the iM. Switching the order of the purine residues to 5′-AG-3′ (i.e. C_3_AGAG and AGAGC_3_) causes significant drop in *T*_m_ by 19°C for purines at the 3′-end (C_3_AGAG versus C_3_GAGA) and by 12°C for the 5′-end (AGAGC_3_ versus GAGAC_3_).
- Neglecting the sequence polarity and other structural effects, presence of the C-G step is slightly less stabilizing than A-C (C_3_GAGA versus GAGAC_3_, both containing (GA)_2_ segment) and G-C is more stabilizing than C-A (AGAGC_3_ versus C_3_AGAG, both containing (AG)_2_ segment). We hypothesize that the presence of the A-C step and (GA)_2_ block is linked to a significantly greater stability of GAGAC_3_ compared to C_3_AGAG with C-A step and a single GA step, cf. Figure 3.

### Structure of i-motif Clip

#### C_3_R_4_ adopts extended form of i-motif

**Figure 4.**
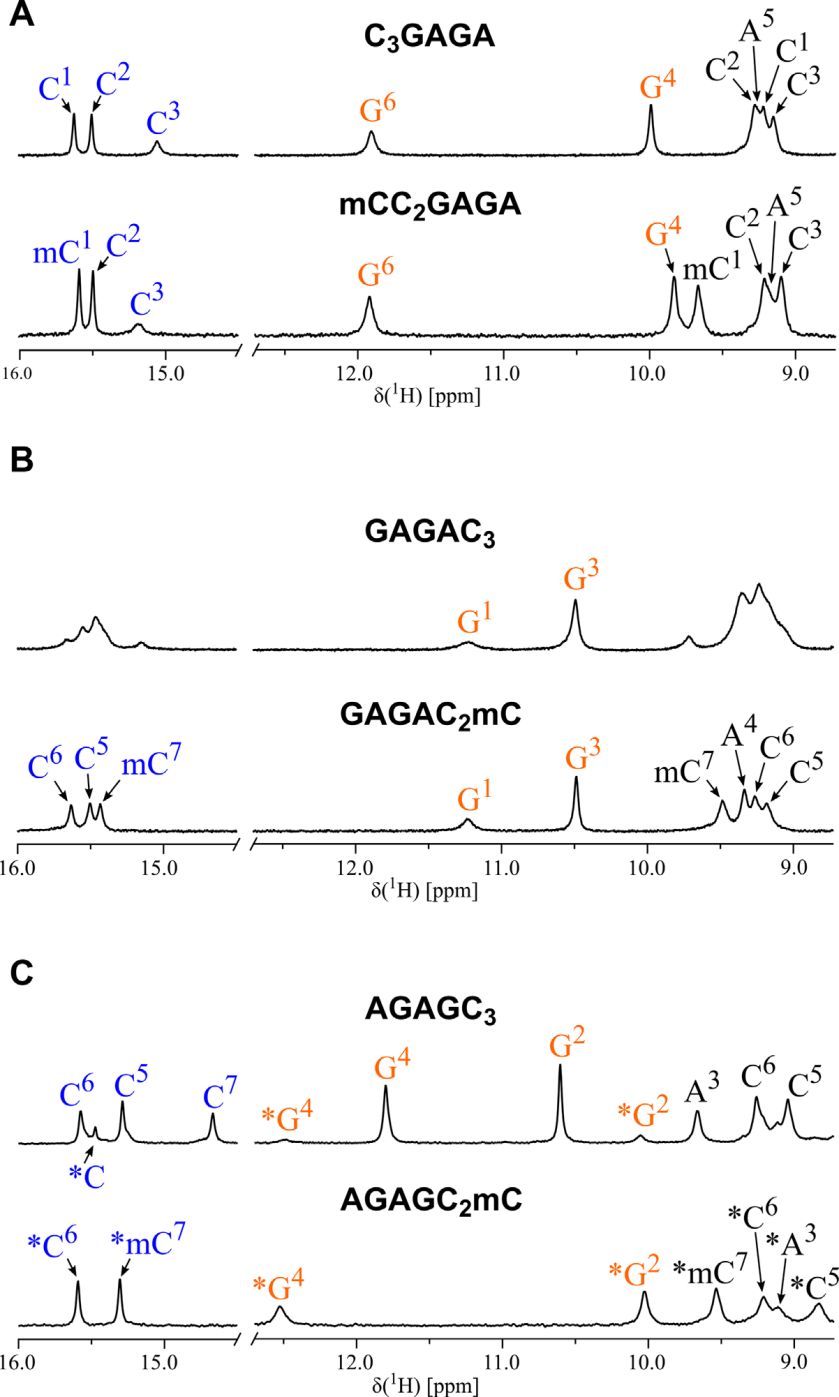
Exchangeable proton region of ^1^H NMR spectra recorded on non-modified and mC-modified sequences (**A**) C_3_GAGA, (**B**) GAGAC_3_ and (**C**) AGAGC_3_ at 5°C and pH ∼5. DNA concentrations per strand were (A) 1.3, 0.9; (B) 1.4, 1.0; (C) 1.2, 0.8 mM. Signals assigned by blue labels correspond to N3H of cytosines, orange signals correspond to guanine N1H and resonances in black refer to NH_2_. Labels with asterisk (*) refer to a structure of an alternative iM topology. For temperature dependance of aromatic ^1^H signals, see Supplementary Figures S14 and S19.

In the ^1^H NMR spectrum of sequence C_3_GAGA one set of cytosine N3H protons was observed (Figure 4A, top, Supplementary Figure S5). Other resonances of cytosine base and sugar protons were assigned based on intra-residual NOE contacts. To distinguish the intercalation topology and associated ambiguous assignment of C^1^ and C^3^ residues, we first focused on inter-duplex contacts (H2′/H2′)*_I_*-(NH_2_)*_II_* present exclusively in the structure of iM (Supplementary Figure S6) (64). Such contacts suggested the extended 3′*E* topology of iM. More detailed discussion of signal assignment using mC modification is given in Supplementary Data (see also Supplementary Figures S5, S7 and S8). Incorporation of mC in the sequence C_3_GAGA (i.e., mCC_2_GAGA and C_2_mCGAGA) has no effect on the topology of iM (Figure 4A, bottom), which is also manifested by the same type of CD spectra showing only a slight increase of the iM band (Supplementary Figure S9). This is also in agreement with an increase of *T*_m_ (∼7°C) caused by mC^1^ and only a minor one (∼2°C) in the case of mC^3^. Similar to C_3_GAGA, no hysteresis between melting and renaturation curves is displayed by the two methylated analogues. The direct stacking between C^3^ and G^4^ bps was confirmed by NOE connectivity (Supplementary Figure S10). Despite poorly resolved NMR signals of C_3_AGAG indicating a labile structure (Supplementary Figure S11), we were able to identify the iM to be in the extended 3′*E* topology.

#### R_4_C_3_ features equilibrium between extended and compact topology of iM

The broadened and overlapped cytosine N3H signals suggest a presence of two interconverting iM topologies (Figure 4B, top, Supplementary Figure S12) in GAGAC_3_. The equilibrium was shifted by mC modification of the 3′-terminal cytosine in the sequence GAGAC_2_mC which adopts the extended *5*′*E* topology (Figure 4B, bottom, see also amplified bands in CD spectrum, Supplementary Figure S13). Methylation of both C^5^ and C^7^ resulted in a significant increase in *T*_m_ by 11 and 9°C, respectively. However, the slope of the CD melting curve of GAGAmCC_2_ is less steep reflecting lower cooperativity of the transition (Supplementary Figure S13), which along with broadened ^1^H NMR signals (Supplementary Figure S12) suggest increased structural variability compared to GAGAC_2_mC. Melting and renaturation curves of GAGAC_3_ display an extensive hysteresis (Supplementary Figure S3), which is distinctly affected by methylation: the hysteresis of GAGAmCC_2_ is significantly reduced whereas that of GAGAC_2_mC is even larger as compared to the parental GAGAC_3_. Based on the retrospective comparison of imino resonances between GAGAC_3_ and GAGAC_2_mC, we assume that the dominant set of signals in GAGAC_3_ corresponds to a structure with iM in extended 5′*E* topology. Because of the reasons indicated above, we focused on the modified GAGAC_2_mC instead of GAGAC_3_.

In ^1^H NMR spectrum of AGAGC_3_, one dominant set of cytosine N3H signals is observed confirming the presence of iM (Figure 4C, top, Supplementary Figure S15). The NOE contacts (Supplementary Figure S6) revealed the compact 3′*E* topology of iM. Upon mC-for-C^7^ substitution in AGAGC_2_mC, the iM completely switched to the extended 5′*E* topology. The perturbed ^1^H NMR shifts (Figure 4C, bottom, Supplementary Figures S16 and S17) and altered pattern of (H2′/H2″)*_I_*-(NH_2_)*_II_* NOE contacts in AGAGC_2_mC compared to AGAGC_3_ further support compact 3′*E* topology in the non-modified AGAGC_3_. In AGAGmCC_2_, the ratio between the compact and extended topologies shifted to ∼1:1, as estimated from the integrals of ^1^H NMR signals. The significant impact of cytosine methylation on the fold of AGAGC_3_ is also demonstrated in CD spectra, where the mC-for-C^7^ substitution (AGAGC_2_mC) caused amplification of the 260 nm band (Supplementary Figure S18). The difference in CD spectral pattern might be linked with the structural rearrangement in purine segment, *vide infra*. Both mC^5^ and mC^7^ substitutions led to an increase in *T*_m_ (∼5–7°C).

### Structure of purine homoduplex

#### Purines in C_3_GAGA adopt four different base-pair geometries whereas those in C_3_AGAG remain mostly unstructured

**Figure 5.**
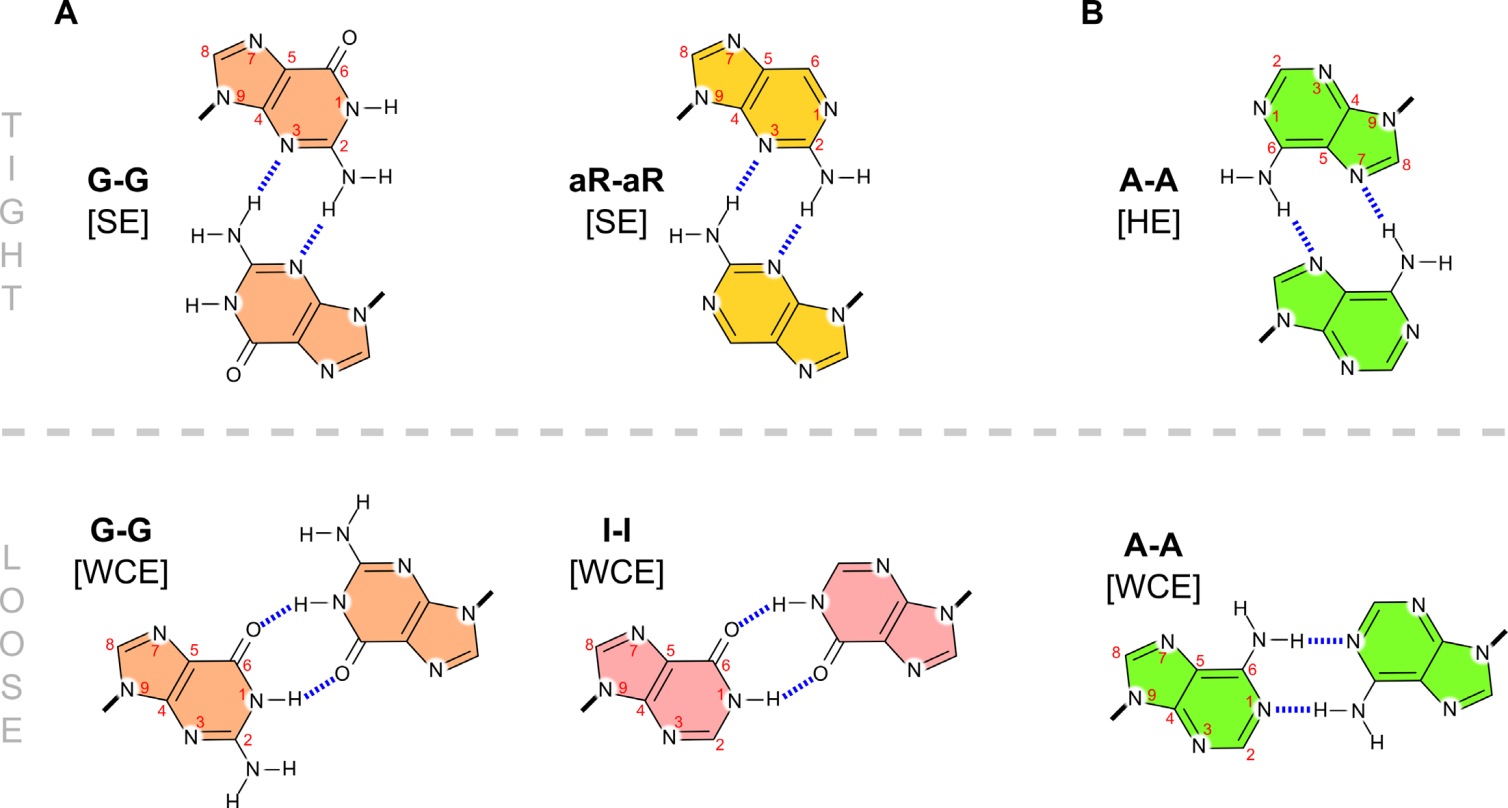
Schematic representation of *C_2_*-symmetrical homopurine base pairing geometries: (**A**) guanine (G), 2-aminopurine (aR), and inosine (I), (**B**) adenine (A). Top row shows tight type of base pairing (SE sugar edge, HE Hoogsteen edge), bottom row—loose arrangement (WCE Watson-Crick edge). Note the distinct separation of glycosidic bonds between tight and loose type of purine pairing. Thus, backbone restraints of neighboring bps represent one of discriminating factors influencing preferred pairing of purine nucleotides.

NOE sequential walk ${\rm{C}}_I^2$-${\rm{C}}_{II}^1$-${\rm{C}}_I^3$-${\rm{G}}_I^4$ established the starting point for an assignment of the remaining part of the purine segment. The standard (H1′/H2′)*^i^*-H8*^i+1^* NOE connectivity was used to assign the signals of aromatic protons. The ^1^H resonance at 10.0 ppm was assigned to G^4^N1H based on its NOE contacts to C^3^H5 and A^5^H1′/H8. A significant shielding of G^4^N1H indicates that it is not participating in any stable H-bond (sugar edge arrangement in Figure 5A, top). Further, we detected the signal of G^4^NH_2_ (at 5.9 ppm) which is crucial for a stabilization of the only possible *C*_2_-symmetrical pairing occurring at the sugar edge (SE). The other signal in the imino ^1^H region at 11.9 ppm belongs to G^6^N1H involved in H-bond. In contrast to G^4^, the G^6^ forms a bp via Watson-Crick edge where the NH_2_ is unbound (Figure 5A, bottom).

To confirm the proposed bp geometries of guanine residues, we carried out substitution experiments exploiting inosine(I)- and 2-aminopurine(aR)-based nucleotides (for ^1^H NMR and CD spectra, see Supplementary Figures S20 and S21, respectively). As expected, the replacement of G^4^ by inosine in C_3_IAGA leads to a dramatic destabilization (decrease in *T*_m_ by 16°C and significant reduction of CD intensity at 240 nm), whereas inosine in C_3_GAIA destabilizes the structure much less (decrease in *T*_m_ by 4°C). Note that C_3_IAGA is the only sequence with iM at the 5′-end, which displays a distinct hysteresis (Supplementary Figure S3). Both ^1^H NMR and CD experiments confirm substantial lability of C_3_IAGA structure. Replacement of G^4^ base by 2-aminopurine (aR) allowed us to examine the situation where only the amino group is present at the pairing interface (see Figure 5A, top). Such sequence adopts well-defined secondary structure only at low temperature (5°C). In the light of all these observations, both N1H and NH_2_ of G^4^ seem to be essential for the formation and stability of purine homoduplex (role of N1H in the SE arrangement is described in section MD perspective). Sequence C_3_AGAG is not capable of forming well-defined structure of purine homoduplex at room temperature (Supplementary Figure S11).

Finally, we were also able to identify the structure of sandwiched A^5^ bp (Figure 5B). Well-resolved A^5^N6H amino proton signals in combination with relatively strong NOE contacts to A^5^H8 suggest a tight HE bp. Despite dynamic opening of the terminal A^7^ bp we were able to determine its propensity to adopt WCE arrangement as determined by dipolar NOE contacts G^6^H8-A^7^H8 and G^6^NH_2_-A^7^H2. The structure of CGA segment in C_3_GAGA shows clear similarity to CGA in TCGA duplex (Supplementary Figure S1) (26).

Extending the purine duplex by additional G in C_3_(GA)_2_G and GAG in C_3_(GA)_3_G did not prevent the formation of the structure of (GA)_2_ block observed in C_3_GAGA. In the case of C_3_(GA)_2_G, a higher structure uniformity was achieved compared to C_3_GAGA because of an additional stabilization of A^7^ bp. In contrast, the additional residues in C_3_(GA)_3_G caused a severe broadening of G^6^N1H suggesting an exchange between alternative pairing modes in longer constructs (Supplementary Figure S22). The CD spectroscopy revealed an increase in amplitude of 260 nm band without significant impact on *T*_m_ (Supplementary Figure S23). The conformation of the sugar-phosphate backbone was mapped in the well-defined structure of C_3_(GA)_2_G using ^1^H-^31^P correlation showing two outlying ^31^P signals assigned to G^4^ and A^5^ residues (Supplementary Figure S24) that represent a transition from iM to GAGA segment (C^3^pG^4^) and a stretched conformation of the backbone connecting nucleotides in the GA step (G^4^pA^5^). Unusual conformation of phosphate linkage was also observed in G·A mismatches both in DNA and RNA antiparallel duplexes (65,66).

#### GA step is formed in R_4_C_3_ regardless of sequential context

**Figure 6.**
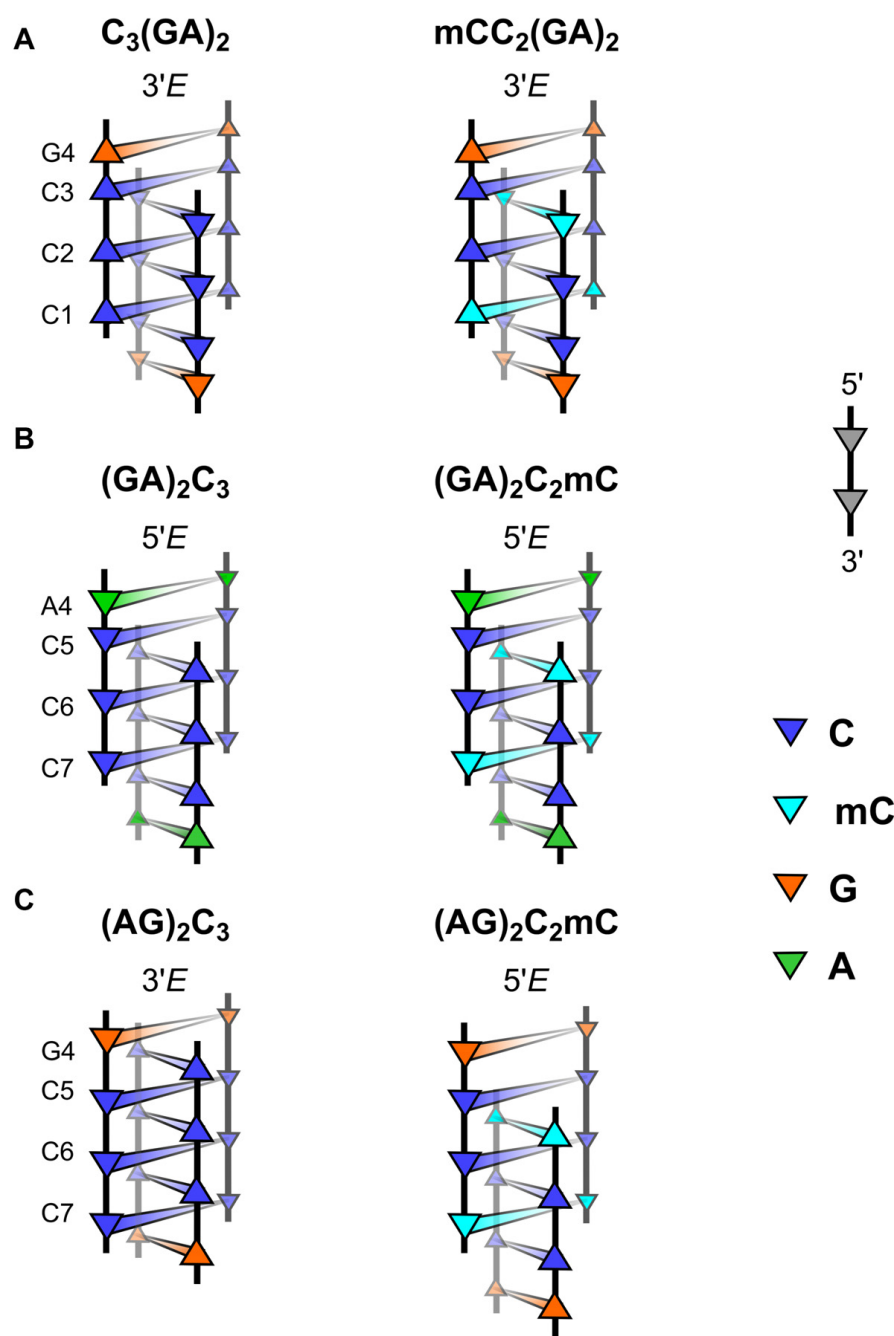
Schematic representation of iM topologies of the unmodified and mC-modified sequences: (**A**) C_3_GAGA (**B**) GAGAC_3_ and (**C**) AGAGC_3_. For clarity, only the iM clip and the neighbouring purine bp are shown. For sequences GAGAC_3_ and AGAGC_3_, only the predominant iM topology is displayed. Complete scheme including minor forms is shown in Supplementary Figure S30.

The broadened NMR lines and low-intensity bands in CD spectra of GAGAC_3_ indicate equilibrium of two iM topologies. The mC-for-C^7^ substitution shifts the equilibrium toward extended 5′*E* iM topology (Figures 4B and Figure 6). The terminal G^1^ nucleotide does not form a stable bp which, subsequently, also affects a formation of the neighbouring A^2^ bp. Assignment of NOESY spectra revealed that G^3^-A^4^ adopts the structure of the GA step, *vide infra*. The A^4^ bp in the tight HE geometry (Figure 5B) stacked on 5′-face of C^5^ bp forms a rigid motif and is probably responsible for high thermal stability of GAGAC_2_mC (Supplementary Figure S25).

In AGAGC_3_, the structure of the stable GA step is formed by G^2^-A^3^ segment. Additionally, the G^4^ adopts *syn*-conformation of the glycosidic bond enabling the formation of the loose WCE G^4^ bp (Figure 5A) which probably facilitates the intercalation of ${\rm{C}}_{II}^7$ bp between ${\rm{G}}_I^4$ and ${\rm{C}}_I^5$ bps and allows formation of the more stable 3′*E* compact topology (as described in the previous section, AGAGC_3_ shows higher propensity to form compact topology of iM). The arrangement of residues at the junction in AGAGC_3_ differs from the one described for A_2_C_4_ (36) in which the iM adopts extended 5′*E* topology and tight A^2^ bp in HE geometry stacks directly on C^3^ bp of iM. The integrity of AGAGC_3_ is almost completely lost upon substitution of inosine for guanosine (Supplementary Figure S26) which supports an essential role of AGAG segment for the overall stability. The base pairing in G^2^-A^3^-G^4^ segment is preserved despite the conversion of iM into extended 5′*E* topology observed in AGAGC_2_mC (Figure 4C). The relative position of guanine N1H in ^1^H NMR spectra remained unchanged, only the difference in chemical shifts is more pronounced (Supplementary Figure S27). Compared to AGAGC_3_, the extended topology of iM in AGAGC_2_mC is associated with residue G^4^ adopting *anti-*conformation of the glycosidic bond and direct stacking of ${\rm{G}}_I^4$ bp on 5′-face of ${\rm{C}}_I^5$ bp (Figure 6). A detailed view on the CG step of both models is shown in Supplementary Figure S28.

#### Conserved structure of GA step

The detailed analysis of NOESY spectra allowed context-independent identification of NOE contacts observed in the conserved GA step and their classification as inter- or intra-strand (Supplementary Figure S29). Additionally, the characteristic values of ^1^H NMR shifts of guanine N1H are preserved in all well-defined structures regardless of the relative position or topology of the iM. If we disregard the presence of the terminal (unpaired) A nucleotide, 5′-**GA***G***-**3′ segment remains, in which 5′-**G** exhibits lower chemical shift of N1H (∼10–11 ppm, SE bp) compared to *G***-**3′ (∼11–12 ppm, WCE bp). The presence of G·G bp at 3′ terminus is not essential for the formation of 5′-**GA**-3′ step. In sequences containing GAGA segment such as C_3_**GA***G*A(G), GA**GA**C_3_, only the GA attached to the iM clip adopts the structure of GA step and prevents formation of another inter-strand stacked GA step in its vicinity. We hypothesize that the impossibility to adopt two consecutive GA steps originates in the poor stacking between A·A bp in HE geometry and G·G bp in SE geometry. Following this hypothesis, we speculate that low thermal stability of sequence C_3_AGAG originates in iM enforcing tight HE geometry of adjacent A^4^ bp which would result in a poor overlap with G^5^ bp thus destabilizing the core of the structure. It is documented in the literature that a preceding nucleotide can influence the pairing of purines in a palindromic antiparallel duplex (65). Interestingly, the bp geometry of G·A mismatch is dictated by the position of purine in the sequence: in d(Y-**GA**-R)·d(Y-**GA**-R) the H-bonds are formed between the sugar edge of G and the Hoogsteen edge of A which results in inter-strand stacking similar to the one described above. In contrast, the Watson-Crick edges of G and A are preferred in G·A mismatches in the d(R-**GA**-Y)·d(R-**GA**-Y) context.

*Two major findings related to our CD and NMR experiments*. First, we described the iM topologies and the effect of mC-for-C substitution on the topology of tetrameric iM in R_4_C_3_ and C_3_R_4_ type of sequences. The iMs of non-modified sequences adopt generally more stable 3′*E* topology except for sequence GAGAC_3_. There are two factors affecting the folding topology: i) the preference of iM to adopt 3′*E* rather than 5′*E* topology and ii) an obstruction of the direct stacking in C-R and R-C steps by an intercalation of CH^+^·C base-pair occurring in the compact topology of iM. In C_3_GAGA adopting extended 3′*E* topology with direct C^3^-G^4^ stacking, the methylation of C^1^ does not change the iM topology as the two effects act in synergy which is responsible for structural uniformity. In the hypothetical compact 5′*E* topology, the mC^1^ bp is intercalated between C^3^ and G^4^ preventing their efficient base overlap that is present in extended 3′*E* topology (Figure 6A). In contrast, in R_4_C_2_mC the 3′*E* topology is compact which prevents direct R^4^-C^5^ stacking and results in the two effects acting in competition. We explain the increased preference for extended 5′*E* topology in R_4_C_2_mC compared to R_4_C_3_ by hindrance of sterically demanding mC^7^ bp intercalated between R^4^ and C^5^ (Figure 6B, C). The mC, sequentially incorporated next to purine in C_2_mCR_4_ and R_4_mCC_2_, seems to prefer the extended iM topology due to a more efficient intra-strand stacking with the R·R base-pair compared to the compact topology. We pointed out the ability of iM to switch the topology to allow a direct intra-strand R-C stacking.

Second, we described the base pairing geometries of purines arranged in the parallel homoduplex and structural changes at the duplex-iM junction induced by the altered iM topology. It has become evident that the base pairing geometry of purines adjacent to the iM clip represents the resultant of structural forces arising from both iM topology and neighbouring purine bps. We described the GA step as a conserved structural motif formed in all well-defined structures. However, our results indicate that the GA step does not simply repeat within a repetitive (GA)_*n*_ segment. In the following section, we rationalize the differences in the thermal stability and the preference of iM in GAGAC_3_ to adopt extended 5′*E* topology by analysis of underlying supramolecular interactions identified from unbiased MD simulation.

### MD perspective: supramolecular interactions forcing the purine pairing

**Figure 7.**
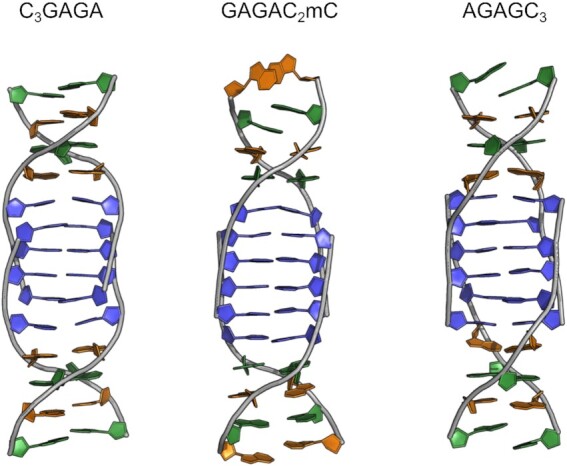
Averaged structures of tetramolecular C_3_GAGA, GAGAC_2_mC, and AGAGC_3_ as obtained from unbiased MD trajectory starting from geometries produced by simulated annealing with NMR restraints (colour coding of nucleotides: C and mC, blue, G, orange, A, green, for technical details, see section Materials and Methods).

The unbiased MD simulations were performed to assess the robustness of the complete tetramolecular models and to rationalize structural peculiarities of the adopted arrangements (Figure 7). The structural rigidity of the most stable sequences C_3_GAGA and GAGAC_2_mC during MD is shown in Supplementary Figures S31 and S32. Despite the rigid iM clips, the purine segment of GAGAC_2_mC exhibits increased flexibility compared to that of C_3_GAGA because of frequent opening of bp at the 5′-end. The tendency to a partial disruption of the structure is even higher in the case of AGAGC_3_ where the intercalated C^7^ bp loses the original structural integrity. Supramolecular interactions (i.e., stacking and hydrogen bonding) detected in MD snapshots from stable time periods were further evaluated with particular attention devoted to the purine homoduplex.

#### Base stacking

**Figure 8.**
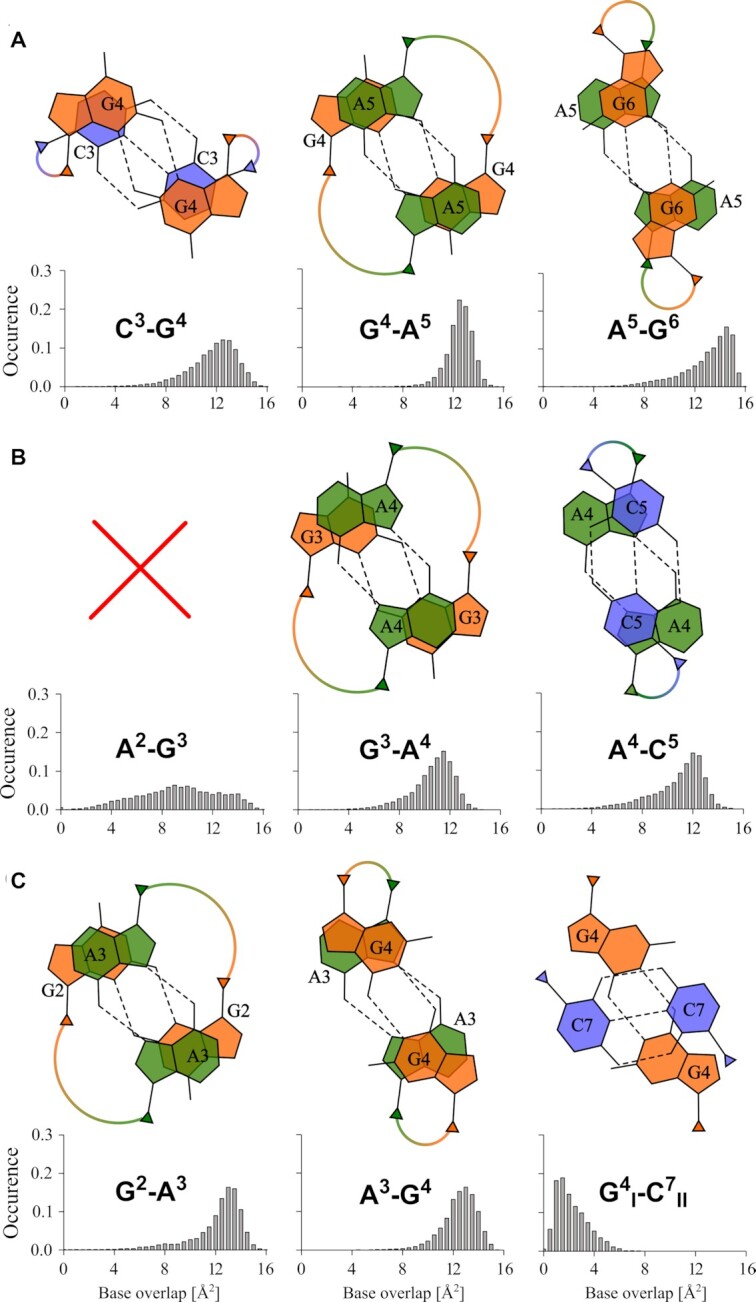
Base-stacking overlaps in averaged structures (**A**) C_3_GAGA, (**B**) GAGAC_2_mC, and (**C**) AGAGC_3_ obtained from 1 μs unbiased MD simulation. The H-bonds are denoted by dashed lines, C1′ atoms by triangles, and helical twists are represented by arcs connecting C1′ atoms. Subscripts *I/II* denote orthogonal duplexes, a red cross indicates absence of single well-defined structure. Note the large helical twist in GA steps. For comparison with AGAGC_2_mC structure, see Supplementary Figure S33. Stacking overlaps were calculated using 3DNA tool (62).

A comparison of base-pair overlaps in sequences C_3_GAGA, GAGAC_2_mC, and AGAGC_3_ is shown in Figure 8. The averaged orientations of stacked nucleobases are in a good agreement with preferences of dinucleotide models simulated in (67). Only the GA step differs significantly from the reported arrangement because of inter-strand character of the base stacking in the parallel duplex described here. For the sequence C_3_GAGA we found that all three consecutive steps C^3^-G^4^, G^4^-A^5^ and A^5^-G^6^ are characterized by substantial bp overlaps of approximately 12 Å^2^. In contrast, only the G^3^-A^4^ and A^4^-C^5^ steps reach comparable overlaps in the sequence GAGAC_2_mC. The step A^2^-G^3^ shows broader distribution of overlap area because of frequent disruptions of A^2^ bp. The stability of AGAGC_3_ structure is disfavoured on an account of a very small overlap of $G_I^4$ with orthogonal $C_{II}^7$ bp. However, the preceding steps G^2^-A^3^ and A^3^-G^4^ are stacked to a similar extend as in sequence C_3_GAGA. From this analysis it is evident that the overlap in the GA step is preserved in presented sequences. Furthermore, the analysis revealed significant difference in base overlap between purine bp and the outermost CH^+^·C bp in extended and compact topology of the iM (Figure 8, compare A^4^-C^5^ and ${\rm{G}}_I^4$*-*${\rm{C}}_{II}^7$). However, the trend in base overlaps is not capable to fully explain the differences in thermal stability (Table 1).

#### Base-backbone hydrogen bonding

**Figure 9.**
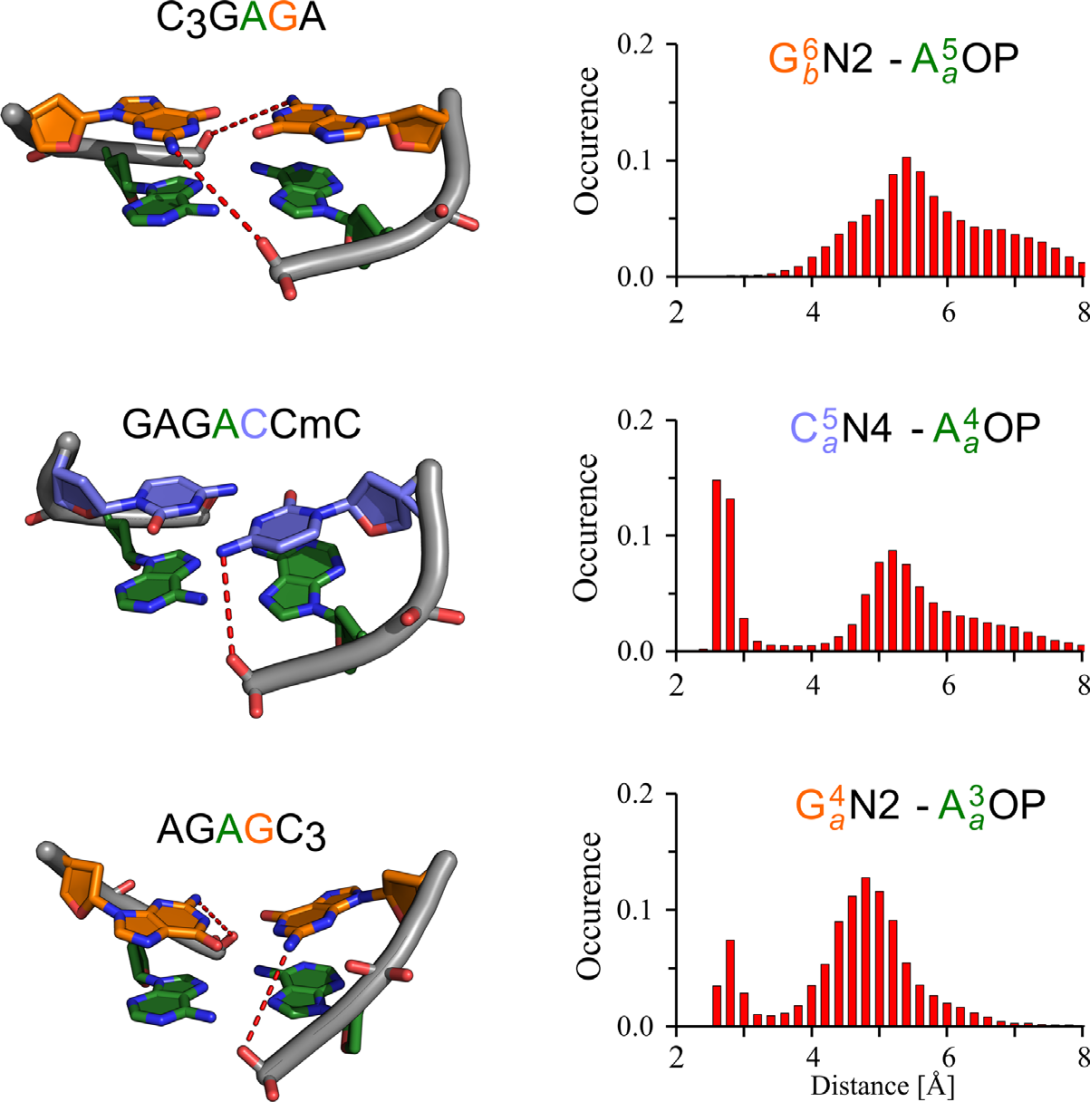
Distance distribution of additional base-backbone hydrogen bonds obtained from 1 μs of unbiased MD performed on complete tetramolecular model. For clarity, only the relevant dinucleotide steps are shown. The H-bonds are denoted in the models with red dashed lines. Labels a/b distinguish the two strands in purine homoduplex. Note the relatively high population of base-backbone H-bond in A^4^-C^5^ step in GAGAC_2_mC stabilizing the backbone curvature and junction between purine homoduplex and iM.

To understand the differences in stabilities we performed an analysis of H-bonds which were extracted from MD trajectory. First, we focused on the base-backbone H-bonds stabilizing the conserved GA step. The inter-strand H-bond between A_a_N6H and A_b_OP was identified already in the structure of TCGA (26). We discovered an intuitive trend in H-bond length distributions governed by relative orientations of the GA step and the rigid iM (Supplementary Figure S34). However, the differences in length distributions are insufficient to explain the differences in the thermal stabilities. We then identified a formation of transient base-backbone H-bonds between phosphate group in the conserved GpA step and the amino group of 3′ adjacent residue with respect to A (G^6^, C^5^ and G^4^ in C_3_**GA**GA, GA**GA**C_2_mC, and A**GA**GC_3_, respectively; Figure 9). The most populated H-bond is formed in GAGAC_2_mC between C^5^NH_2_ and A^4^OP group within one strand stabilizing the homoduplex-iM junction. An analogous inter-strand H-bond in C_3_GAGA between G^6^NH_2_ and A^5^OP is disabled because of a larger separation of the strands in the purine homoduplex. Metastable G^4^ bp in AGAGC_3_ structure is shaped mainly by intra-residual NH_2_-OP interaction which probably depopulates the remote contact with A^3^OP. The importance of G^4^NH_2_-G^4^OP interaction for the stability was demonstrated on the sequence AGAIC_3_, in which the absence of NH_2_ group prevented formation of a stable secondary structure. Thus, we suspect that relatively high melting temperature of GAGAC_2_mC originates in the GAC region. Despite the structural flexibility of G^1^ and A^2^ residues, the large A^4^-C^5^ base overlap enforced by C^5^NH_2_ bridges with a phosphate group seems to be the key factor imparting a greater thermal stability to the core region of this sequence. Base-backbone H-bonds are also known to be important for stability of G·A mismatches in sheared geometry formed in tandem (68).

#### Supramolecular interactions at iM-homoduplex junction described using MD

Complementing our experimental data by theoretical approach allowed us to clarify the preference of iM for the 5′*E* topology in GAGAC_3_ by presence of additional base-backbone H-bonds and efficient base stacking in A-C step located at homoduplex-iM junction. We were able to pinpoint interatomic contacts explaining the trend in thermal stabilities. We showed that presence of sequence-dependent weak supramolecular interactions can shift the equilibrium from generally preferred 3′*E* to 5′*E* topology. We described sequence C_3_GAGA in which the efficiency of base-stacking and favourability of 3′*E* iM topology act in synergy resulting in structural uniformity and thermal stability of both non-modified and mC-modified sequence. We highlighted the structural differences at iM-homoduplex junction in sequences AGAGC_3_ and AGAGC_2_mC. We determined that the G-C step is the most versatile as the G was observed to adopt *syn*-conformation to accommodate orthogonal CH^+^·C base-pair in compact 3′*E* topology whereas in extended 5′*E* topology the *anti*-conformation is preferred.

### Summary

To summarize our work, we determined the structure of d(GA) repeats arranged in parallel duplex enforced by iM clip. We described a conserved structural motif adopted by 5′-GA-3′ block (termed GA step), formed independently on the position of iM, and showed that GA step does not simply repeat in the parallel d(GA)_n_ segment. Additionally, we showed that 5-methylation of cytosine affects the equilibrium between 3′*E* and 5′*E* topology of tetrameric iM in a sequence-dependent manner and we described the changes at the junction between duplex and iM induced by the altered topology of the iM clip. Finally, we rationalized the roles of different base pairing geometries and their steric requirements, efficiency of base-pair stacking, and formation of transient base-backbone H-bonds on the stability. We described two non-canonical structural motifs, which not just coexist in a single supramolecular arrangement but even significantly stabilize individual building blocks. The only analogous study reported a simultaneous existence of i-motif and G-quadruplex in a single molecule (63). Unprecedented structural details revealed in this study provide valuable insights into the structure–stability relationship which are applicable in other non-canonical arrangements of nucleic acids.

## DATA AVAILABILITY

Atomic coordinates have been deposited in the Protein Data bank under accession numbers: *7BI0* (C_3_GAGA, 10 snapshots + averaged structure from 1 μs MD trajectory), *7BL0* (GAC_2_mC stable part, 10 snapshots + averaged structure from 1 μs MD trajectory), *7BLM* (AGAGC_3_: 3 snapshots + averaged structure from initial 0.3 μs part of MD trajectory), *7BMA* (AGAGC_2_mC 10 snapshots + averaged structure from 1 μs MD trajectory). Summary of experimental restrains and statistical parameters calculated for initial models and MD snapshots is reported in Supplementary Table S1A, B.

## Supplementary Material

gkab915_Supplemental_File
