# Supplementary material for: Revealing structural peculiarities of homopurine GA repetition stuck by i-motif clip
Source: Nucleic Acids Res. 2021 Oct 28;49(20):11425–37. doi: 10.1093/nar/gkab915 (PMC8599794; doi:10.1093/nar/gkab915)
Supplement: gkab915_Supplemental_File [file gkab915_supplemental_file.docx]

SUPPLEMENTARY DATA

Revealing Structural Peculiarities of Homopurine GA Repetition Stuck by i-Motif Clip

Aleš Novotný,^†,‡^ Jan Novotný,*^,†,‡^ Iva Kejnovská,^$^ Michaela Vorlíčková,^$^ Radovan Fiala,^†,‡^ and Radek Marek*^†,‡^

*^†^ CEITEC – Central European Institute of Technology, Masaryk University,*

*Kamenice 5, CZ-62500 Brno, Czechia*

*^‡^ National Centre for Biomolecular Research, Faculty of Science, Masaryk University, Kamenice 5, CZ-625 00 Brno, Czechia*

*^$^ Institute of Biophysics of the Czech Academy of Sciences, Královopolská 135, CZ-612 65 Brno, Czechia*


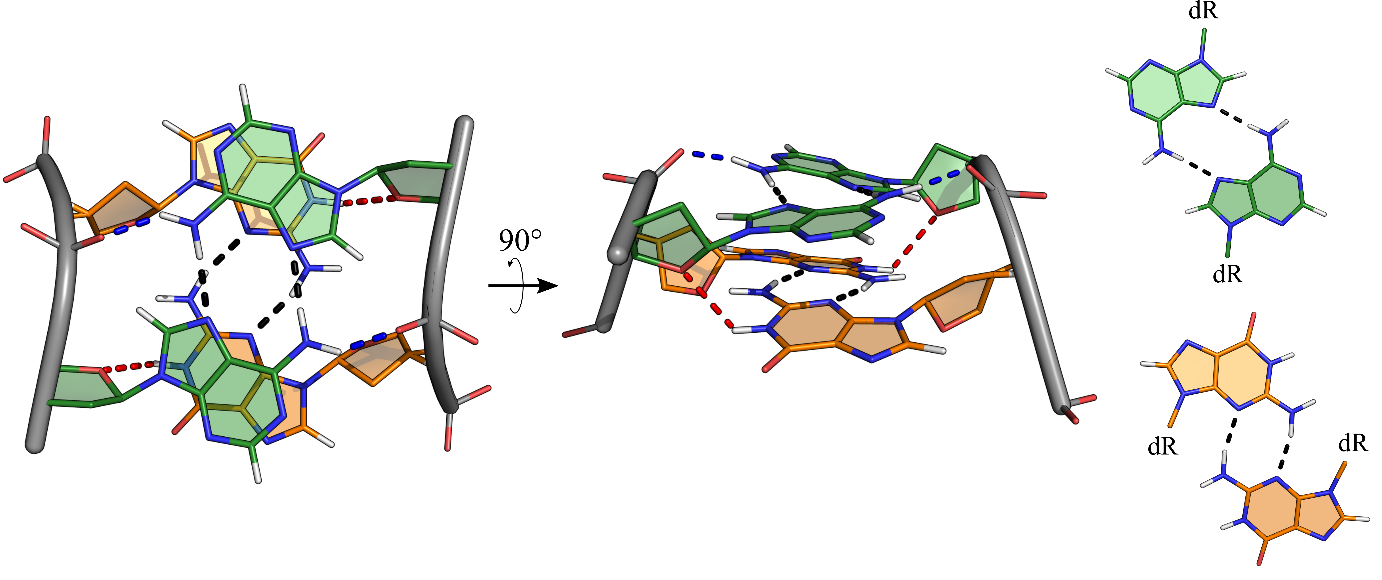


Figure S1. The GA step extracted from published NMR-derived structure of a parallel homoduplex TCGA (top and side view) (1). Guanine pair (shown in orange) in anti-orientation of glycosidic bond adopts sugar-edge pairing (SE). Adenine residues (green) are paired via Hoogsteen edge (HE). Note additional weak hydrogen bonding of amino/imino-protons with backbone of the opposite strand (depicted with red and blue dashed lines).


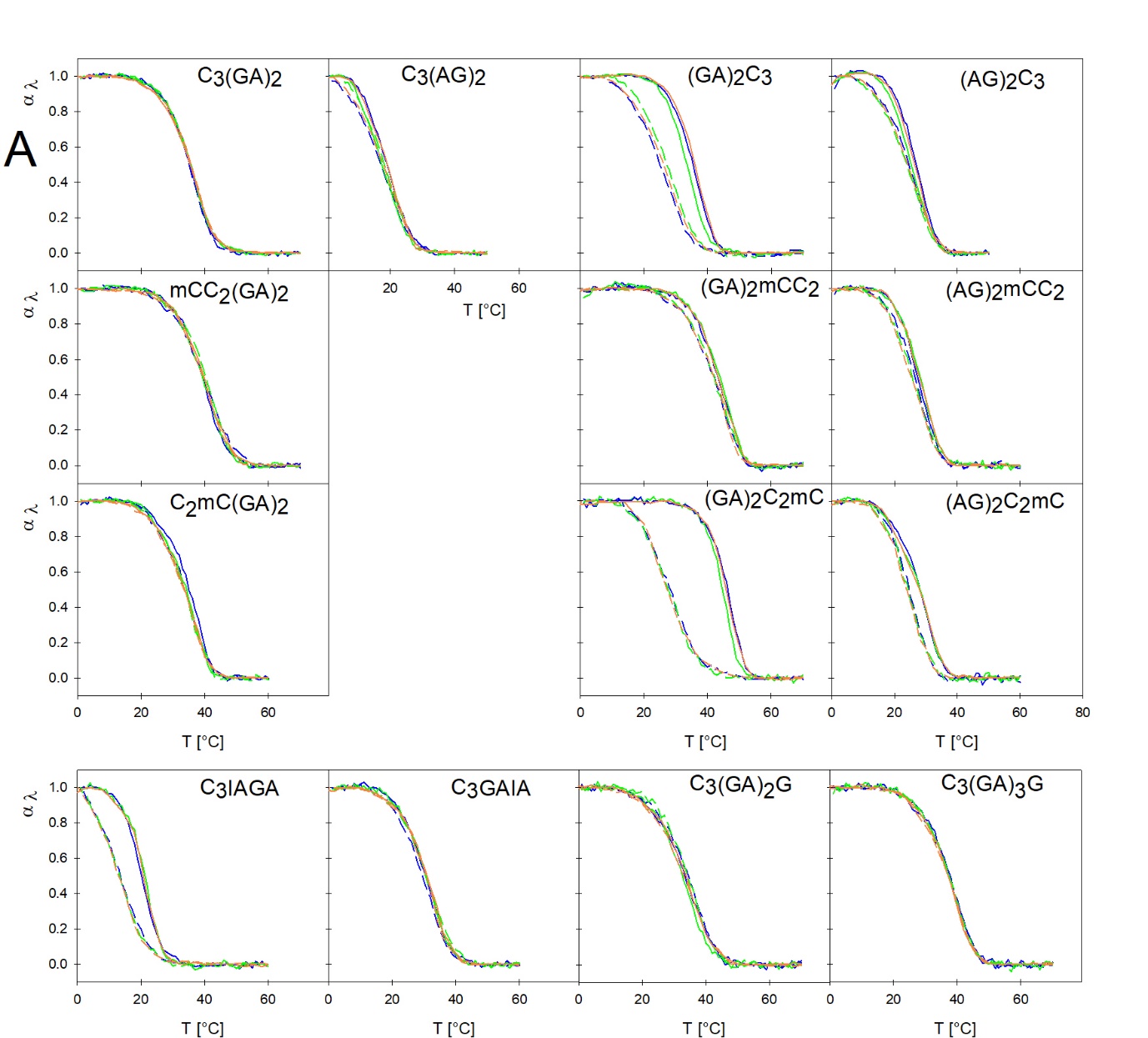


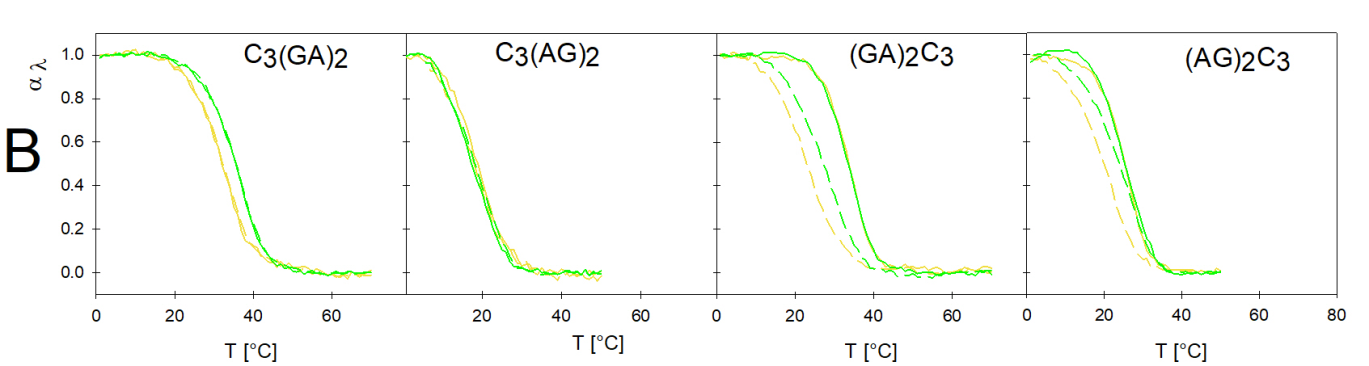


Figure S3. Temperature dependences of C_3_GAGA, C_3_AGAG, GAGAC_3_ and AGAGC_3_ and their modified analogues measured in 65 mM K^+^, pH 5, at a DNA strand concentration of 0.15 mM.

**A:** Melting (full lines) and renaturation (dashed lines) experiments measured at three rates of temperature changes: 0.33 (red), 0.22 (blue) and 0.13 (green) °C/min.  Oligonucleotides with iM on the 5´-end display no hysteresis between their melting and annealing curves. The only exception was C_3_IAGA. The hysteresis of its temperature dependences seems to be related to a huge destabilization and heavy refolding caused by the substitution of its G^4^ by inosine.

In contrast, the oligonucleotides with iM on the 3´-end display significant hysteresis. It is especially pronounced with (GA)_2_C_3_ as compared to (AG)_2_C_3_, while its extent is still increased with C^7^ methylation in the case of (GA)_2_C_2_mC but nearly no hysteresis is observed with its C^5^ methylation. Decreasing the rate of temperature changes has only very slight effect on the extent of hysteresis. This indicates that even the fastest temperature changes used (0.33°C/min) were sufficient for equilibrating the sequences at particular temperatures, and that the hysteresis is mainly a result of their distinct melting and refolding kinetics.

**B:** The course of temperature dependences (solid lines - melting, and dashed lines - renaturation) recorded in 1°C temperature intervals at a rate of temperature changes 0.13^o^C/min in 10 mM potassium phosphate and 50 mM KCl (65 mM K^+^), adjusted by 0.1 M HCl to pH 5, i.e. in the solution used throughout in all experiments in the paper (green),  was compared, for the four main studied sequences, with those measured under the same conditions but in the Robinson-Britton buffer (K-RB), at pH 5 with  KOH added up to 65 mM K^+^ (yellow). The course of temperature dependences is very similar or identical in both solutions. This indicates that the pH value of the unbuffered solution does not significantly change at increased temperatures. Hysteresis is, however, slightly larger in the buffer.


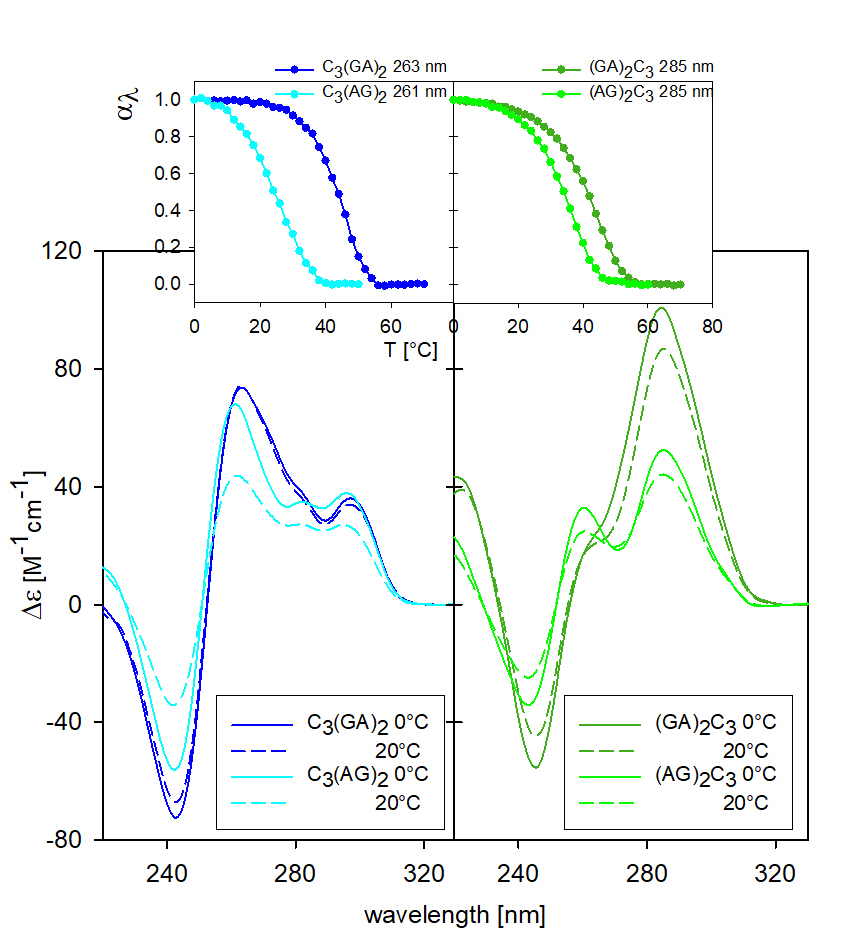


Figure S4. CD spectra and temperature dependences of the studied sequences measured in 65 mM K^+^, pH 5 at 0.7 mM DNA concentration.


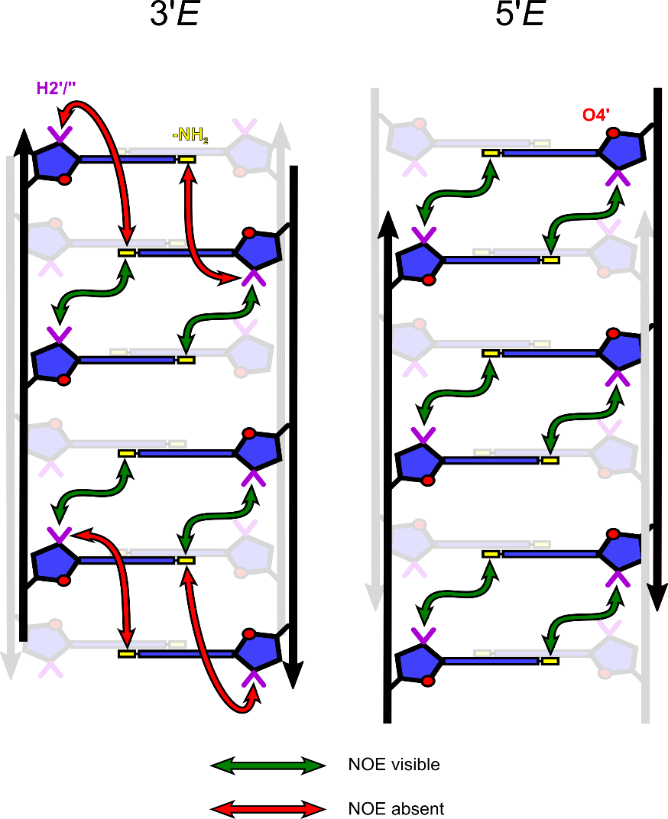


Figure S6. Schematic of NOE contacts within iM used for discrimination of its topology.

To confirm the adopted topology of sequence **C_3_GAGA** and to complete the assignment of ^1^H NMR spectra, we employed 5-methylation of C^1^ (**mCC_2_GAGA**) and C^3^ (**C_2_mCGAGA**). The introduction of mC did not cause significant changes in the structure and confirmed original 3’*E* topology of the iM (Figure S5). External position of C^3^ was documented by several C^3^-G^4^ contacts (e.g., Me/H41-N1H, H1’/H2’’-H8 - Figure S10).


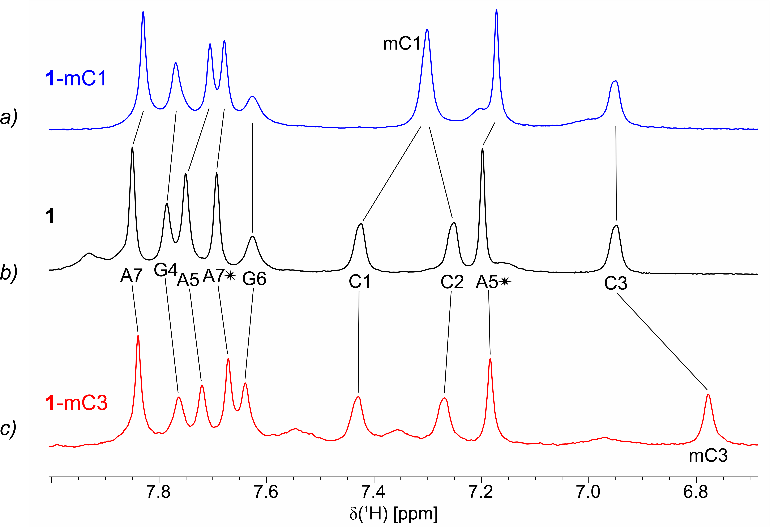


Figure S7. Aromatic and amino regions of ^1^H NMR spectra of *a)* sequence **mCC_2_GAGA** (0.9 mM), *b)* **C_3_GAGA** (1.3 mM) and *c)* **C_2_mCGAGA** (0.6 mM) at 5°C. Non-exchangeable protons H8 and H6 are assigned according to residue labels. Resonances A^5^H2 and A^7^H2 are marked with asterisk.

**
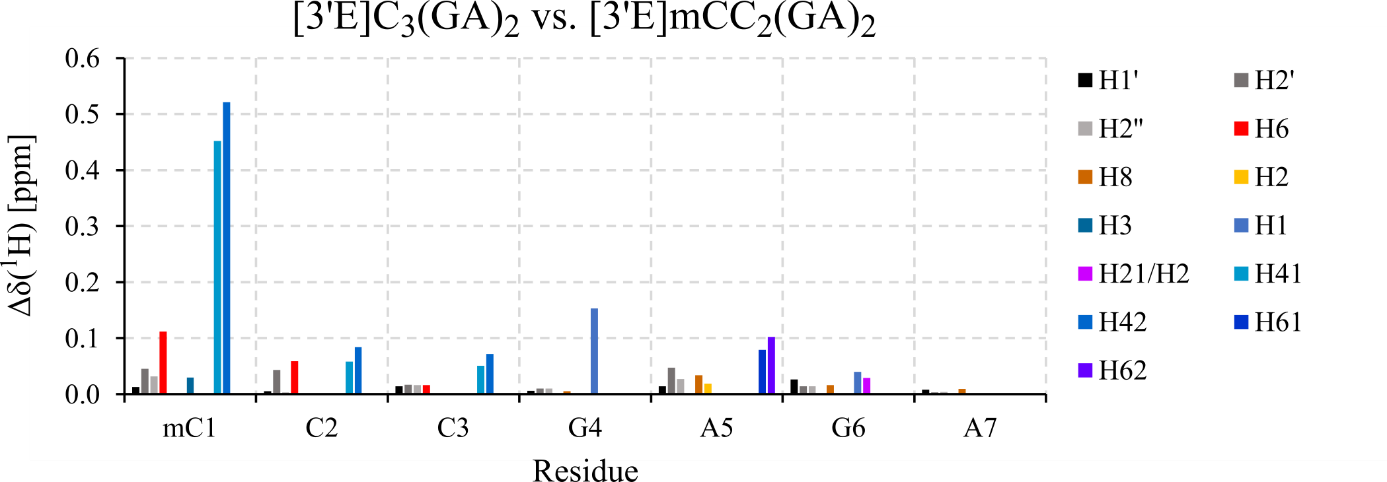
**

**
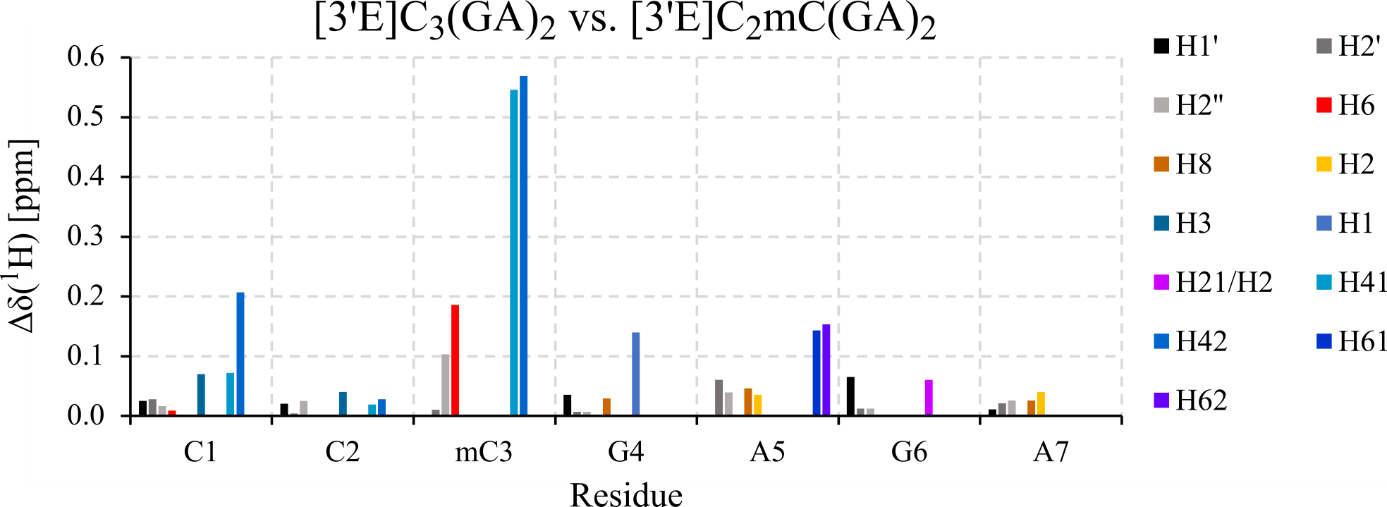
**

Figure S8. Absolute values of chemical shift differences reflecting the effect of cytosine methylation on ^1^H NMR resonances of **mCC_2_GAGA** and **C_2_mCGAGA** relative to **C_3_GAGA**.


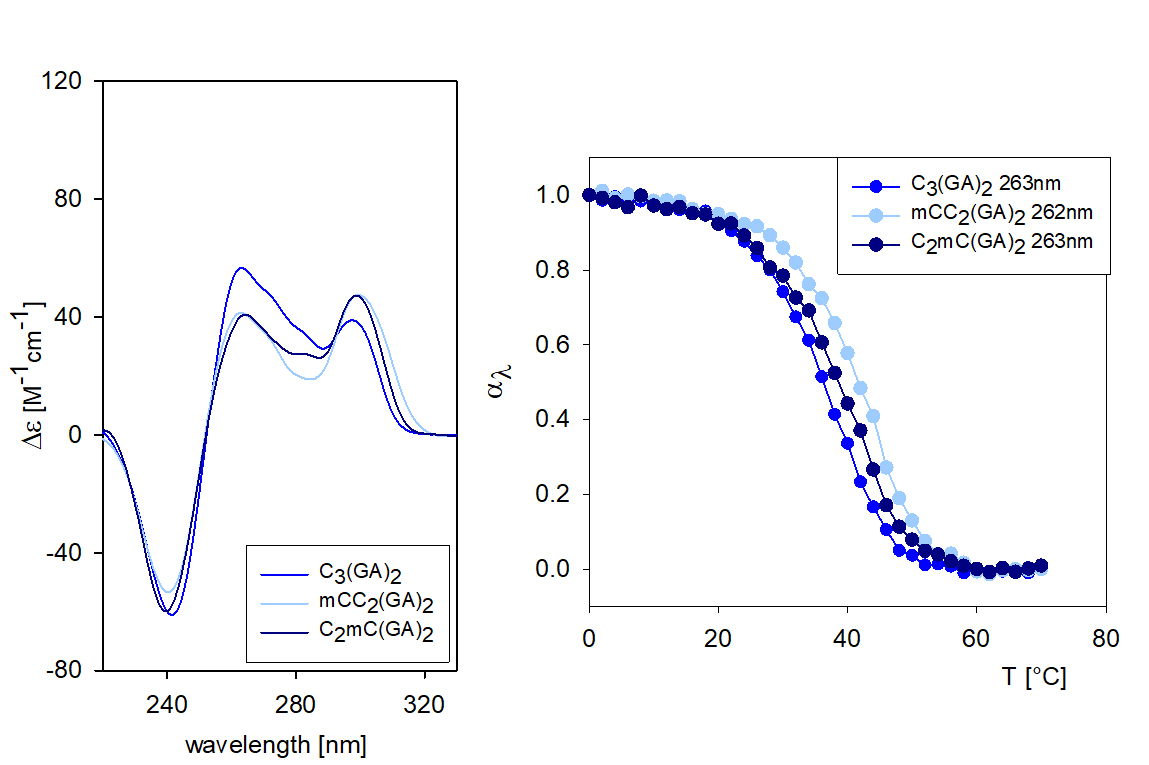


Figure S9. Effect of C^1^ and C^3^ methylation of sequence **C_3_GAGA**: CD spectra (left) and temperature dependences (right) measured in 65 mM K^+^ at 0.15 mM DNA concentration. In accord with literature data, C-methylation stabilizes the resulting structures of both C_3_GAGA and GAGAC_3_. Methylation of C^3^ and C^1^ increased T_m_ of C_3_GAGA by 2°C and by 7°C, respectively. Both methylations caused very similar changes in CD: they increased the band corresponding to i-motif and decreased the band at 260 nm.

**
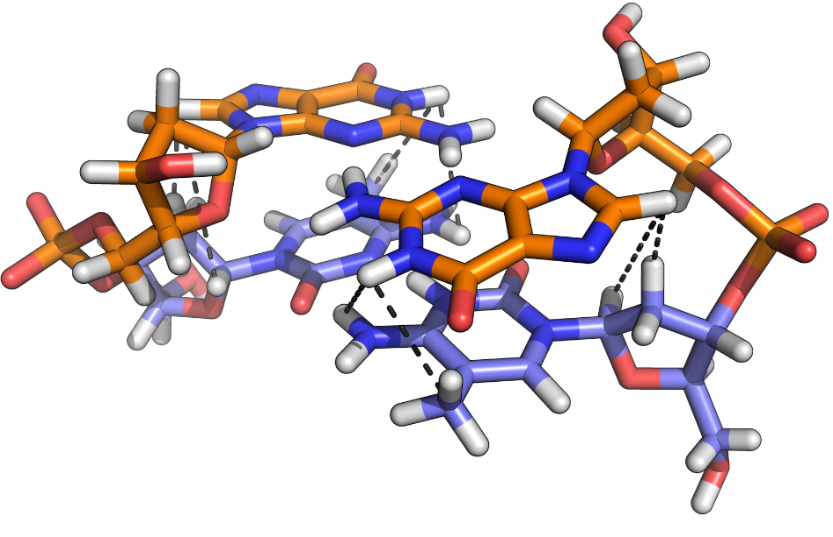
**

Figure S10. C^3^-G^4^ step of model of **C_2_mCGAGA** sequence with depicted vertical NOE contacts confirming extended 3’*E* topology.


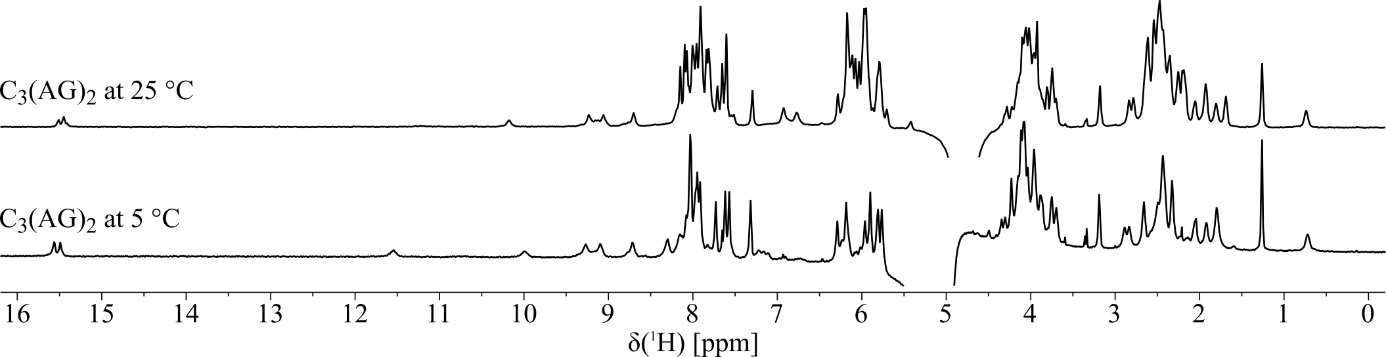


Figure S11. ^1^H NMR spectra of sequences **C_3_AGAG** (1.4 mM) in 50mM KCl/10mM K-phosphate at pH 5 recorded at 5 and 25°C. Note the missing signal of guanine N1H (at 11.5 ppm) at the room temperature.


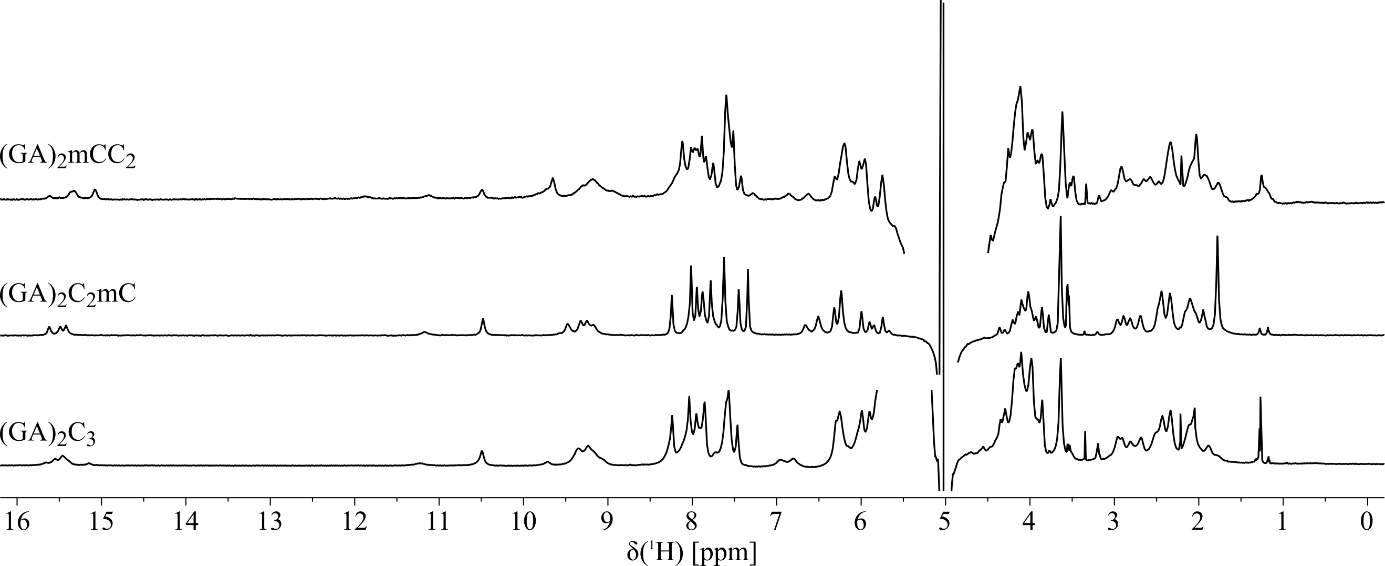


Figure S12. ^1^H NMR spectra of **GAGAC_3_** (*bottom,* 1.4 mM) and its terminally methylated forms **GAGAC_2_mC** (*middle,* 1.0 mM) and **GAGAmCC_2_** (*top,* 0.6 mM) measured at 2°C in 50mM KCl/10mM K-phosphate at pH 5. Note the sharper signals and lower degree of signal overlap in the spectrum of **GAGAC_2_mC** due to the shift of equilibrium towards single iM fold. The exchange broadening remains significant in the **GAGAmCC_2_** and the population of the minor form of iM is increased.


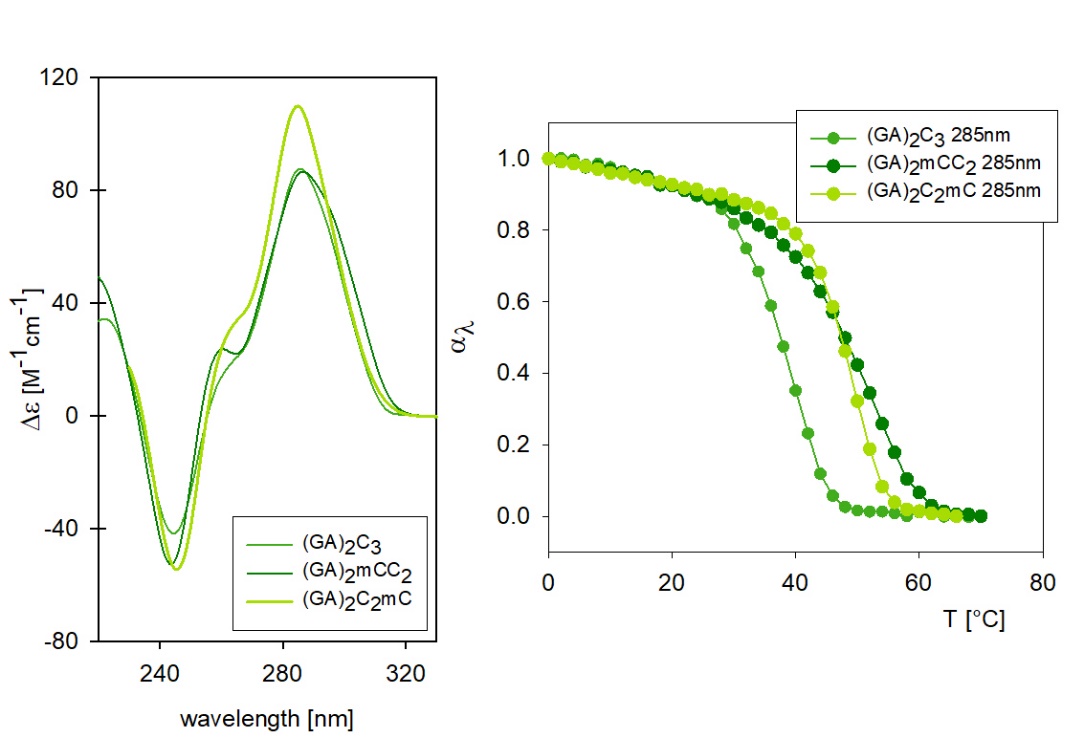


Figure S13. Effect of C^5^ and C^7^ methylation in sequence **GAGAC_3_**. CD spectra (left) and temperature dependences (right) measured in 65 mM K^+^ at 0.15 mM DNA concentration. The sequence was stabilized by methylation more distinctly than its C_3_GAGA counterpart. This may be related to the shift in topological equilibrium of iM. Both methylated analogues exhibit similar thermostability, however, **GAGAmCC_2_** melts less steeply than **GAGAC_2_mC**, which indicates the presence of multiple conformational states. This observation may confirm an isomerization process between different structures, similarly to behavior observed for unmethylated structure of **GAGAC_3_**.


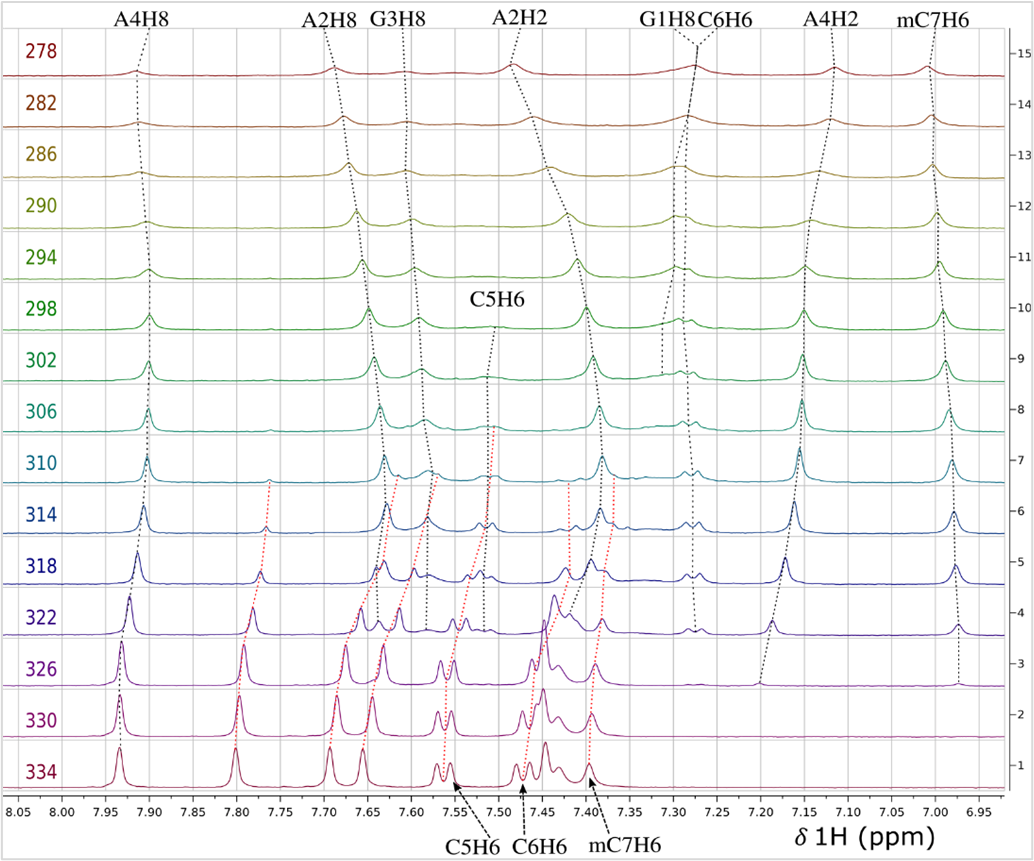


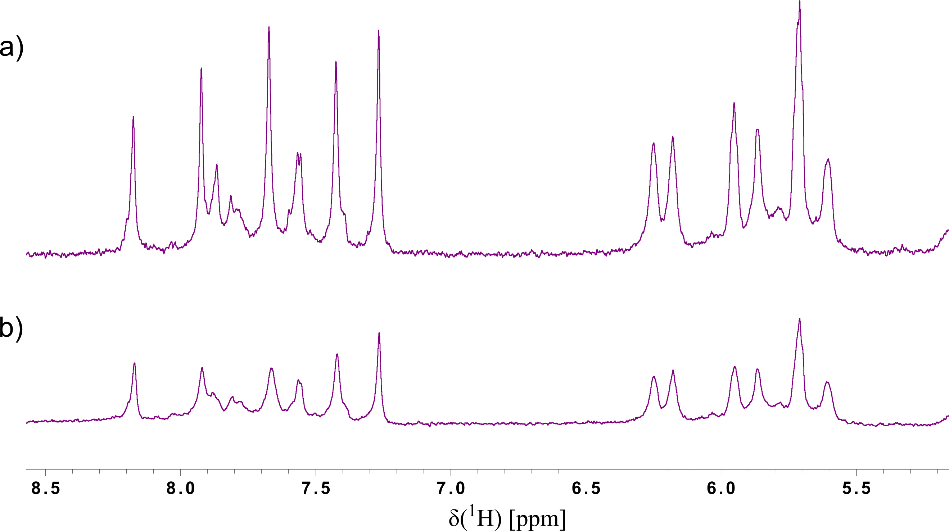


Figure S14. Top: Temperature dependent ^1^H NMR spectra of **GAGAC_2_mC** (strand concentration 1.0 mM, 65 mM K^+^, pH 5). Black lines connect signals of folded species, red lines indicate unfolded single strand species. Inflection occurs around 47°C (320 K). Bottom: Effect of sample dilution from 1.0 mM in a) to 0.5 mM in b) on ^1^H NMR signals of aromatic and anomeric protons in **GAGAC_2_mC**. Note preserved position of all resonances.


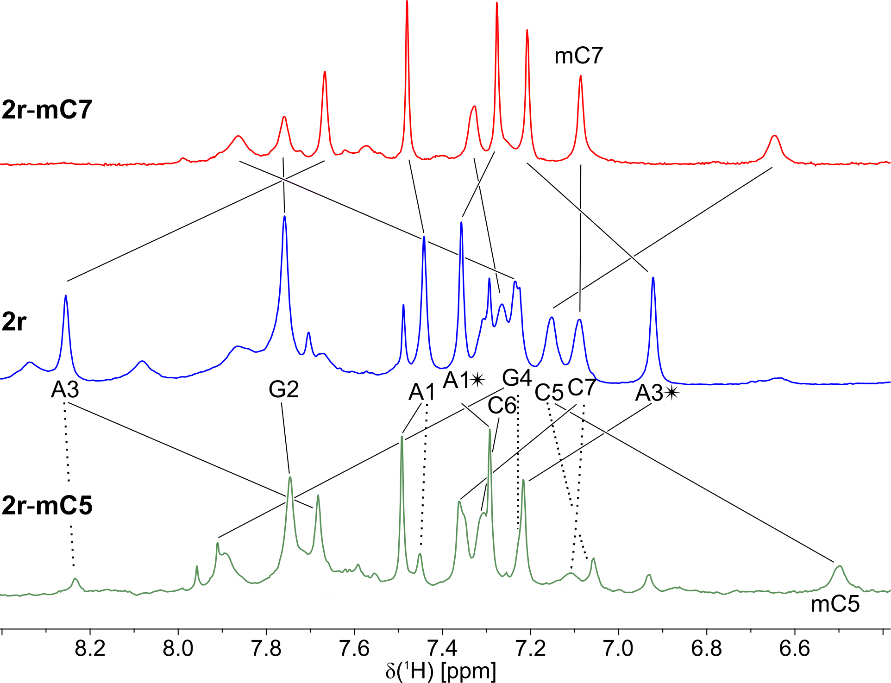


Figure S16. Comparison of aromatic region of ^1^H NMR spectrum of sequence **AGAGC_3_** (*middle*) and its methylated analogues at position C^7^ (*top*) and C^5^ (*bottom*) at 5°C. Only nonexchangeable protons H8 and H6 are labelled with the residue numbers. H2 resonances of adenine nucleobase are marked with asterisk. For simplicity, connecting lines are shown only for resonances exhibiting greatest change of chemical shift upon cytosine methylation. Dashed lines correspond to original 3’*E* topology whereas solid lines represent transition towards 5’*E* topology. Note that shielding response of mC^5^ (-0.6 ppm) in **AGAGmCC_2_** sequence includes synergic effect of methylation and location of mC^5^ on the outside of iM in the extended topology. In contrast, deshielding of C^7^ reflects its relocation to the central part of iM. For **AGAGC_2_mC** oligonucleotide, consistent trends are observed: C^5^ is strongly shielded (-0.4 ppm) because of its external position in the 5’*E* topology whereas the signal of C^7^ does not move because of compensation of the opposing effect of chemical modification (shielding) and conversion of iM topology resulting in relocation of mC^7^ to the central part of iM.


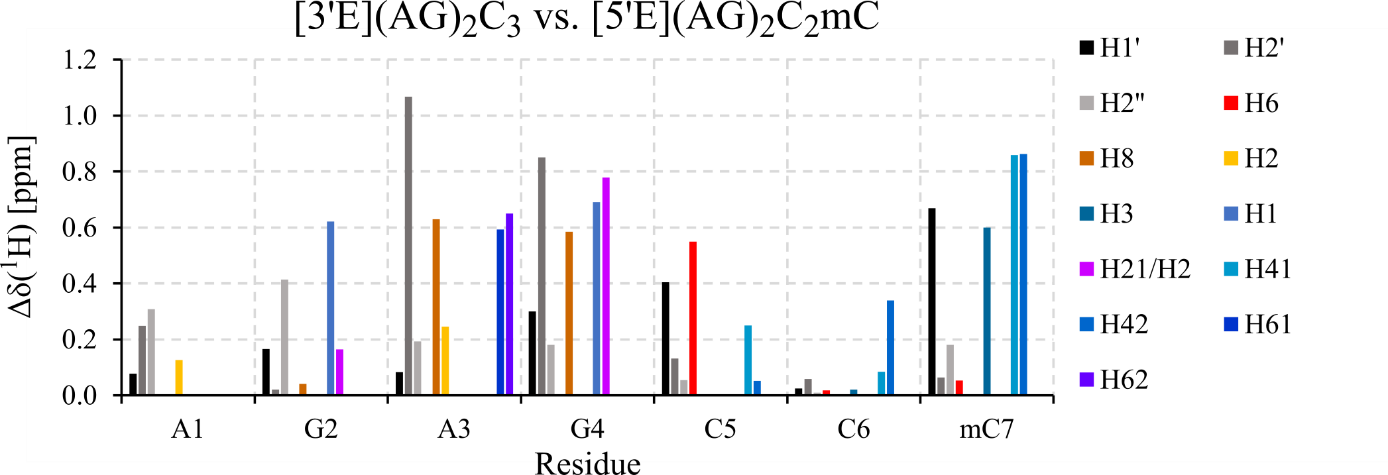


Figure S17. Absolute values of chemical shift differences reflecting the effect of cytosine methylation on ^1^H NMR resonances of **AGAGC_2_mC** relative to **AGAGC_3_**. The complete conversion from compact (3’E) to extended (5’E) topology associated with syn-anti transition of glycosidic bond of G^4^ is manifested by significant changes of chemical shifts in all residues except for the terminal A^1^ and central C^6^.


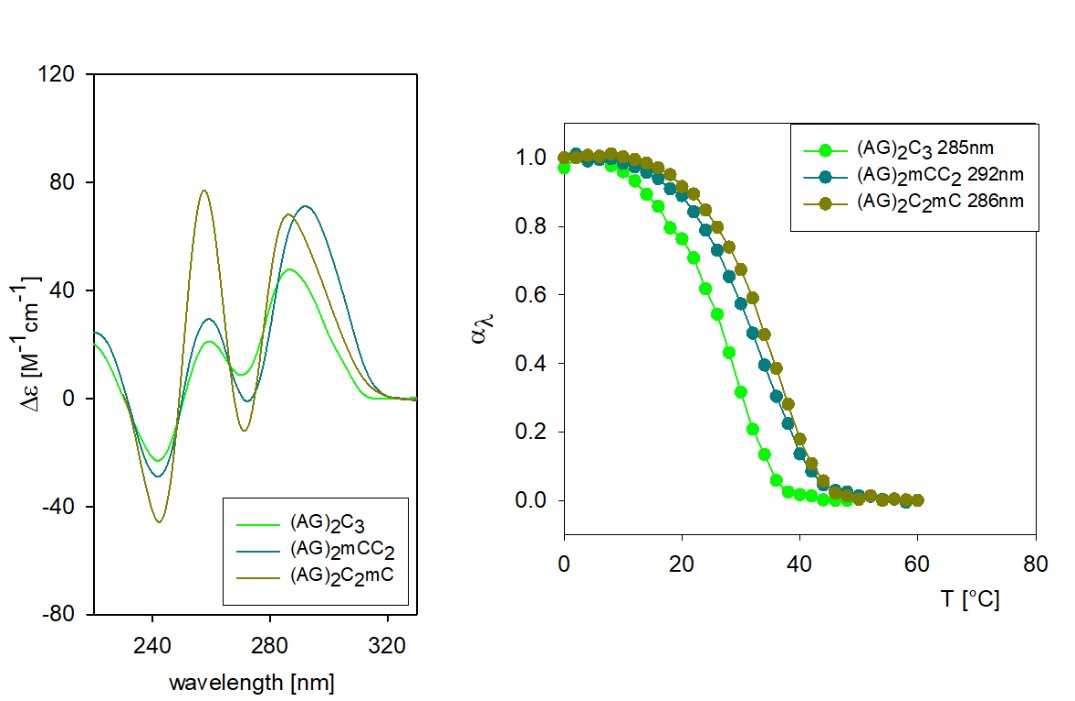


Figure S18. Effect of C^5^ and C^7^ methylation on **AGAGC_3_**: CD spectra (left) and temperature dependences (right) measured in 65 mM K^+^ at 0.15 mM DNA concentration. The **AGAGC_3_** sequence provides CD spectrum of substantially lower amplitudes than **GAGAC_3_**, with clearly resolved long-wavelength positive bands. Both cytosine methylations result in higher T_m_ and a significant increase of iM related band. In addition, the presence of mC^7^ is associated with a sharp amplification of 260 nm band. Thus, **AGAGC_2_mC** shows unusual spectral pattern, which is linked to total conversion of compact iM topology to the extended one.


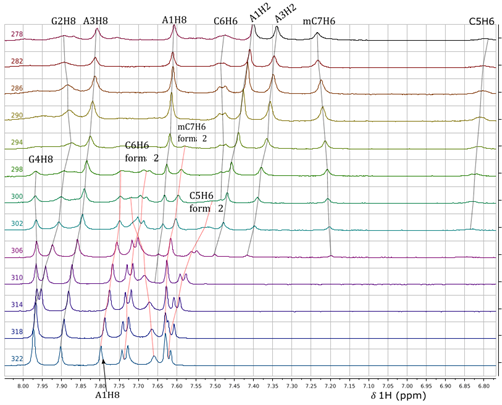


Figure S19 Temperature dependent ^1^H NMR spectra of **AGAGC_2_mC** (c = 0.8 mM, 65 mM K^+^, pH 5). Black lines connect signals of folded species, red lines denote unfolded single strand species. Inflection occurs around 30°C (303 K).


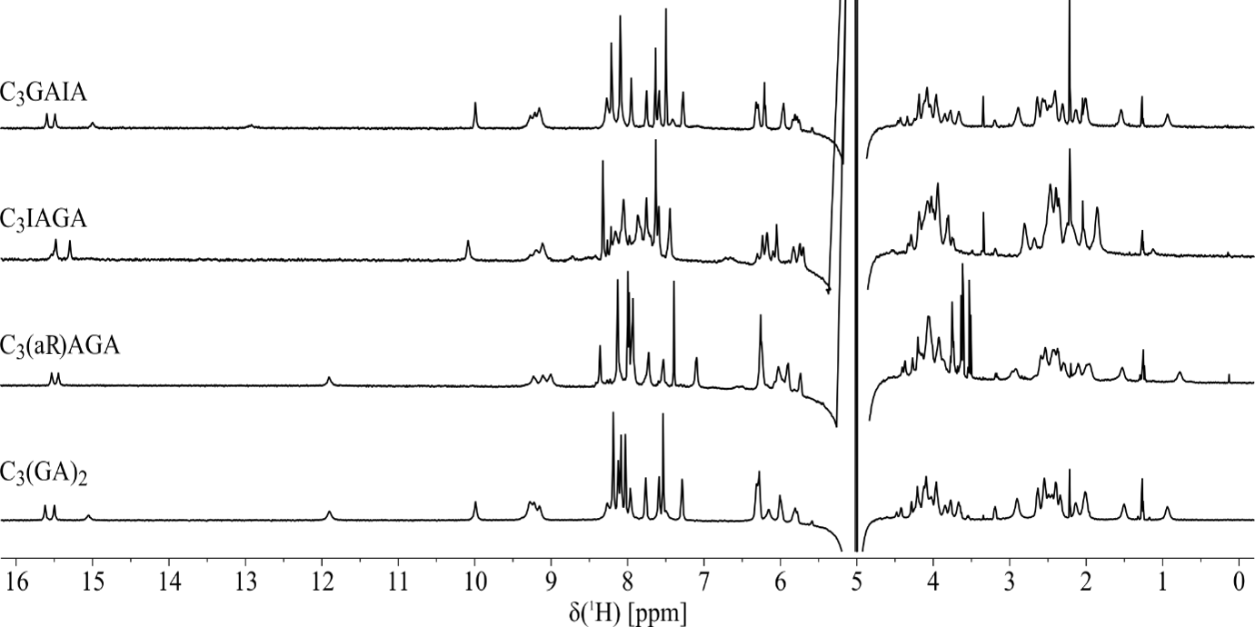


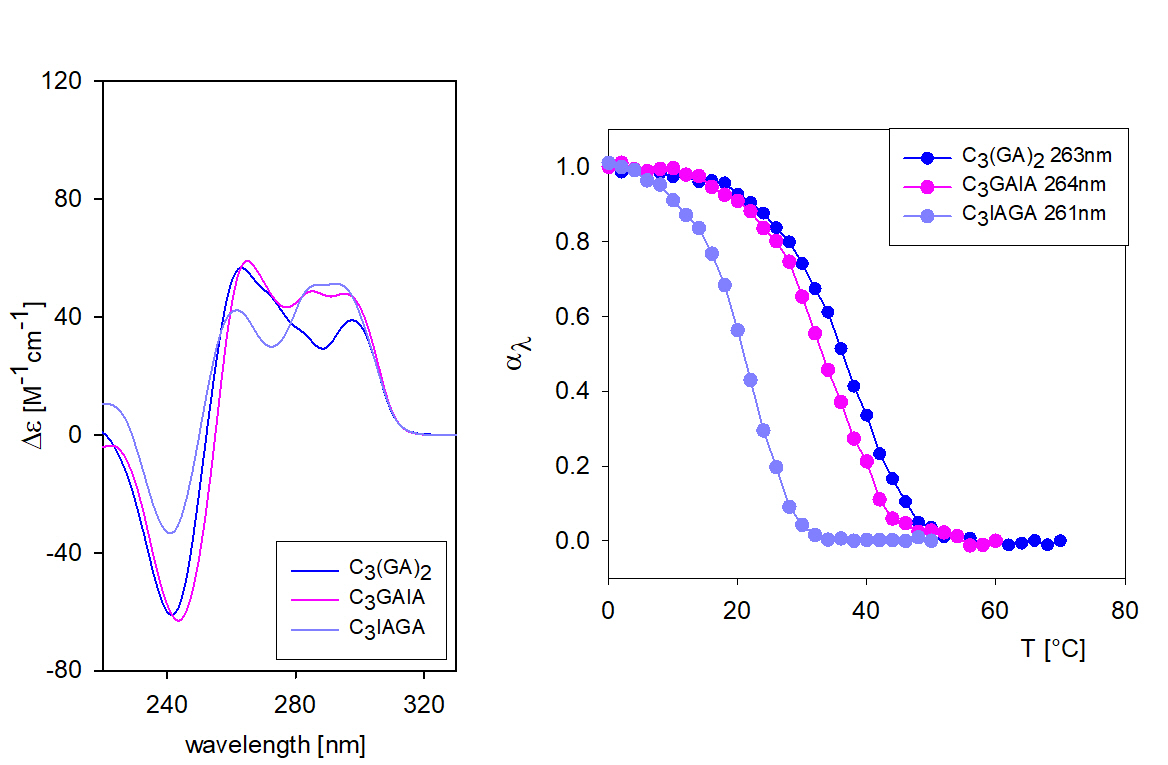


Figure S21. Effect of guanine-hypoxanthine substitution on CD spectra of sequence **C_3_GAGA**. CD spectra (left) and temperature dependences (right) measured in 65 mM K^+^ at 0.15 mM DNA concentration. Note how replacement of G^4^ with I^4^ reduces CD amplitudes at 240 and 260 nm and dramatically destabilizes the overall structure.

It is well documented in literature that longer (GA)_n_ repetitive sequences do not adopt a uniform ensemble of conformers (2, 3), making thus determination of detailed structural features using NMR experiments impossible. Despite these serious limitations we attempted to investigate (GA)_2_ block extended by G and GAG overhangs. A comparison of 1D ^1^H NMR spectra is shown in Figure S22. In general, the structure of octameric **C_3_GAGAG** resembles the fold of original sequence **C_3_GAGA** (documented by identical 3’*E* topology, similar G^4^ and G^6^ chemical shifts and identical set of NOE connectivities). The unaltered position of G^6^ N1H protons implies that the adopted pairing is not affected by a stabilization of the subsequent A^7^. Based on detected NOE contacts 6NH_2_-H2, A^7^ prefers extended WCE (6NH_2_-N1) pairing geometry similarly to preceding G^6^. Note that the signal of terminal G^8^ imino proton was identified at the chemical shift that is approximately average value of G^4^ and G^6^ resonances and is naturally affected by bp fraying.


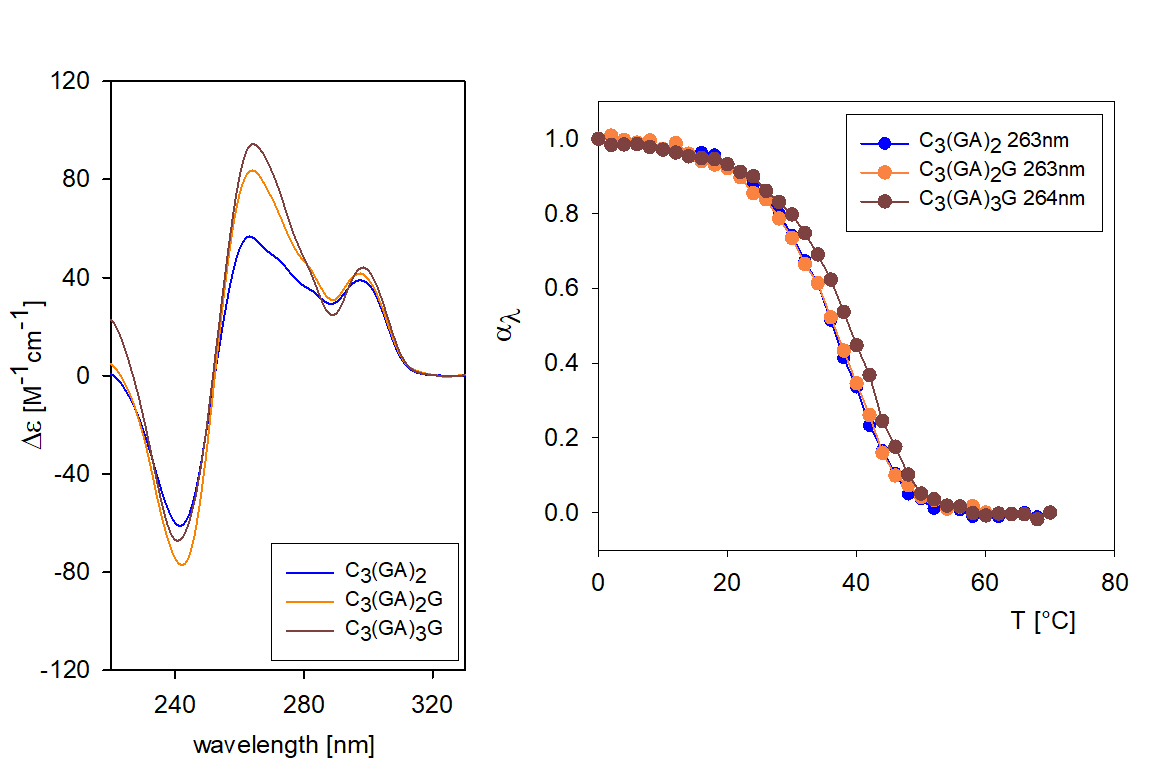


Figure S23. Effect of extension of GA repetition: CD spectra (*left*) and temperature dependences (*right*) measured in 65 mM K^+^ at 0.15 mM DNA concentration. Appended G^8^ or G^8^A^9^G^10^ residues do not change significantly the thermostability of the sequences, which seems to be governed by two strong inner GA steps. The only noticeable change is an increase in the 260 nm band because of the dominating role of the GA component.

A higher level of structure uniformity of **C_3_GAGAG** was responsible for a better quality of NMR spectra compared to **C_3_GAGA**. Therefore, more extensive assignment of NMR signals including ^31^P was performed. ^1^H-^31^P correlation (Figure S24) revealed that C^3^-G^4^ and G^4^-A^5^ adopt non-canonical backbone conformation characterized by deviated shielding values of ^31^P NMR signals (G^4^: -2.2 ppm, A^5^: -0.1 ppm). The rest of the purine sequence exhibits a usual set of correlations in narrow range (-1.2±0.2 ppm).

In contrast, 1D ^1^H NMR spectrum of the sequence **C_3_(GA)_3_G** shows severe broadening of signals of guanine imino protons while the linewidths of imino protons of CH^+^∙C bp in the iM remain nearly unaltered (Figure S22). Chemical shifts of all resolved exchangeable protons stay at a very similar position indicating a pairing pattern similar to that of **C_3_GAGA**. Unfortunately, considerable signal overlaps did not allow any conventional 2D NMR experiments to be analysed.


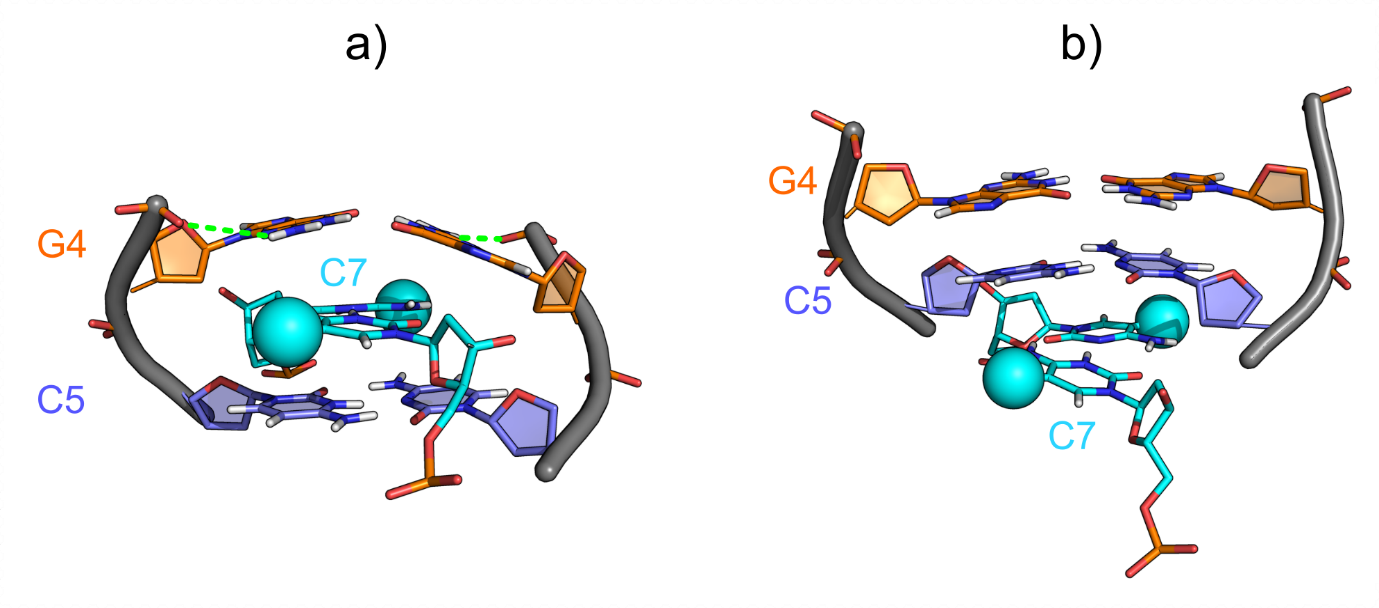


Figure S28 Comparison of G^4^mC7/C5 steps in compact 3’E (a) and extended 5’E (b) topology of iM in sequence **AGAGC_2_mC**. Note the bulged geometry of G^4^ bp in syn-conformation associated with steric requirement of the intercalated mC^7^ in compact topology (the methyl group is represented by a sphere).


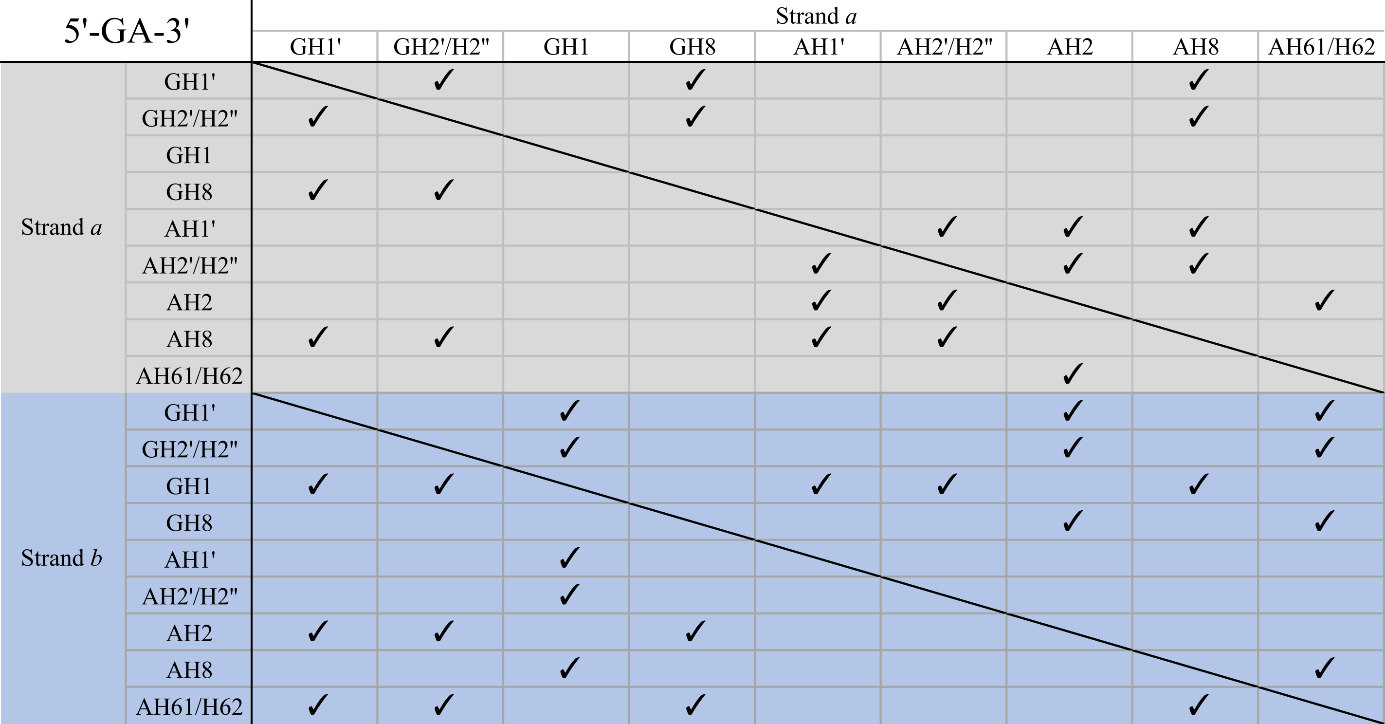


Figure S29 Intra-strand and inter-strand NOE contacts observed in GA step.

Figure S31. Time evolution of RMSD in Å of iM and GAG segments of molecule **C_3_GAGA** extracted form unrestrained MD trajectory. The structure is stable on 1 μs timescale. Torsion angles comprising phosphorus atom adopt their canonical states except for *epsilon* and *zeta* of A^3^. All purine nucleotides adopt C2’-*endo* sugar pucker and *anti*-conformation around the glycosidic bond. A comparison of the CGA segment structure with the average structure of **C_3_GAGA** and TCGA ensemble yielded maximum RMSD of 1.6 Å. Most of the differences arise from the presence of iM on 5’-side of CGA segment in **C_3_GAGA** and T^1^ residue in TCGA, respectively.

Figure S32. Time evolution of RMSD in Å of C_3_ and AGA segments of molecule **GAGAC_2_mC** extracted form unrestrained MD trajectory. Note large fluctuations of the AGA segment due to the base-pair instabilities of 5’-terminal region.

| **Sequence** | **C_3_GAGA** | **GAGAC_2_mC** | **AGAGC_3_** | **AGAGC_2_mC** |
| --- | --- | --- | --- | --- |
| Total number of NOEs | 538 | 734 | 524 | 620 |
| Intra-residual NOEs | 116 | 144 | 128 | 132 |
| Others | 422 | 590 | 396 | 488 |
| Total number of torsions (CHI) restrains | 28 | 28 | 28 | 28 |
| DISTANCE VIOLATIONS | 22 | 66 | 33 | 53 |
| Average/standard deviation [Å] | 0.15 ± 0.06 | 0.16 ± 0.07 | 0.18 ± 0.09 | 0.16 ± 0.08 |
| Maximum violation [Å] | 0.36 | 0.37 | 0.43 | 0.45 |
| TORSION VIOLATIONS* | 0 | 3 | 0 | 2 |
| Number of violations higher than 30° | 0 | 0 | 0 | 0 |

* Average and standard deviation of torsion violations were not calculated due to low number of violations.

Table S1A Overview and statistics of experimental restraints used in simulated annealing to build the initial models.

| **Sequence**, PDB code | **C_3_GAGA** 7BI0* | **GAC_2_mC** 7BL0 | **AGAGC_3_** 7BLM* | **AGAGC_2_mC** 7BMA* |
| --- | --- | --- | --- | --- |
| Number of models in the ensemble | 10 | 10 | 3 | 10 |
| RMSD total | 1.92 ± 0.58 | 1.43 ± 0.26 | 1.20 ± 0.20 | 1.53 ± 0.30 |
| RMSD i-motif | 1.12 ± 0.31 | 1.19 ± 0.22 | 0.95 ± 0.20 | 1.03 ± 0.19 |
| RMSD duplex | 1.71 ± 0.50 | 1.27 ± 0.37 | 1.19 ± 0.28 | 1.39 ± 0.33 |
| DISTANCE VIOLATIONS  in the whole ensemble | 453 | 871 | 156 | 714 |
| Average/standard deviation [Å] | 0.45 ± 0.41 | 0.69 ± 0.64 | 0.80 ± 0.86 | 0.60 ± 0.62 |
| Maximum violation [Å] | 2.66 | 3.55 | 3.49 | 4.30 |
| Number of violations higher than 0.5 Å | 124 | 405 | 73 | 259 |
| TORSION VIOLATIONS  in the whole ensemble | 5 | 12 | 4 | 14 |
| Average/standard deviation [Å] | 4.8 ± 3.2 | 8.0 ± 5.3 | 4.9 ± 5.4 | 6.6 ± 5.6 |
| Maximum violation [Å] | 9.8 | 16.7 | 12.9 | 18.5 |

* Values calculated for all residues except of terminal bp of duplex. Only heavy atoms were considered in the calculation of RMSD values.

Table S1B Overview and statistics of experimental restraints calculated on MD-derived ensemble of models deposited in PDB database. Note that the simulations were restrain-free, in the explicit solvent at 300 K.

BIBLOGRAPHY

1. Wang,Y. and Patel,D.J. (1994) Solution structure of the d(T-C-G-A) duplex at acidic pH. A parallel-stranded helix containing C+ .C, G.G and A.A pairs. *J. Mol. Biol.*, **242**, 508–526.

2. Rippe,K., Fritsch,V., Westhof,E. and Jovin,T.M. (1992) Alternating d(G-A) sequences form a parallel-stranded DNA homoduplex. *EMBO J.*, **11**, 3777–3786.

3. Kejnovská,I., Tůmová,M. and Vorlı́čková,M. (2001) (CGA)4: parallel, anti-parallel, right-handed and left-handed homoduplexes of a trinucleotide repeat DNA. *Biochim. Biophys. Acta*, **1527**, 73–80.
